# Drawing from name in semantic dementia reveals graded object knowledge representations in anterior temporal lobe

**DOI:** 10.3758/s13421-024-01578-9

**Published:** 2024-05-22

**Authors:** Tanmay Anand, Karalyn Patterson, James B Rowe, Thomas E Cope

**Affiliations:** 1 Cambridge University Hospitals NHS Trust, Cambridge, CB2 0QQ UK; 2 Medical Research Council Cognition and Brain Sciences Unit, University of Cambridge, Cambridge, CB2 7EF UK; 3 Department of Clinical Neurosciences, University of Cambridge, Cambridge, CB2 0SZ UK

**Keywords:** Semantic dementia, Semantic memory, Anterior temporal lobe, Drawing, Qualitative observation

## Abstract

Semantic dementia (SD) is characterized by progressive impairment in conceptual knowledge due to anterior temporal lobe (ATL) neurodegeneration. Extended neuropsychological assessments can quantitatively demonstrate the semantic impairment, but this graded loss of knowledge can also be readily observed in the qualitative observation of patients’ recall of single concepts. Here, we present the results of a simple task of object drawing-from-name, by patients with SD (*N* = 19), who have isolated atrophy of the ATL bilaterally. Both cross-sectionally and longitudinally, patient drawings demonstrated a pattern of degradation in which rare and distinctive features (such as the hump on a camel) were lost earliest in disease course, and there was an increase in the intrusion of prototypical features (such as the typical small ears of most mammals on an elephant) with more advanced disease. Crucially, patient drawings showed a continuum of conceptual knowledge loss rather than a binary ‘present’ or ‘absent’ state. Overall, we demonstrate that qualitative evaluation of line drawings of animals and objects provides fascinating insights into the transmodal semantic deficit in SD. Our results are consistent with a distributed-plus-hub model of semantic memory. The graded nature of the deficit in semantic performance observed in our subset of longitudinally observed patients suggests that the temporal lobe binds feature-based semantic attributes in its central convergence zone.

**Supplementary information:**

The online version contains supplementary material available at 10.3758/s13421-024-01578-9.

## Introduction

It is widely accepted that semantic concepts in the human brain correspond to distributed representations across multiple cortical regions (Nastase et al., 2017). However, the way in which these neural representations are bound together into a single multidimensional concept is controversial. Contemporary theories regarding the representation of semantic knowledge have mostly been characterized by one of two different frameworks: a ‘distributed-only’ view, and a ‘distributed plus hub’, or ‘hub-and-spokes’ model (Guo et al., 2013; Lambon Ralph et al., 2017; Martin, 2007; Patterson et al., 2007). This distinction reflects a key question about semantic memory—is there a central convergence zone, or hub, linking the various sensory and perceptual modality-associated cortices that process and represent semantic concepts? If so, what is the mechanism by which this convergence zone performs its function in the representation of, and/or access to, semantic memory?

According to distributed-only models, the association cortices responsible for the higher-level processing of sensory stimuli that form the basis of semantic memory are connected to one another, without the need for a central convergence zone (Martin, 2007). When a concept is relevant to a person’s cognitive state, the components of the concept are each accessed in their respective cortices. This leads to a tiled pattern of activation that links all such regions, recreating the concept in its entirety. Association cortices are interconnected and this population-level neural activation is proposed to be enough for activation of the concept (Huth et al., 2016; Rogers & Lambon Ralph, 2022). The alternative model posits a hub, which acts as a central convergence zone linking the regions that represent the concept’s various perceptual and linguistic components. Activation of a concept relies on the integrity of the hub and its connections to other cortical regions (Guo et al., 2013).

Semantic dementia (SD) provides the opportunity to observe the mechanisms of concept representation. It is a neurodegenerative disease characterized by a progressive impairment in semantic memory. SD is typically associated with aggregation of TDP-43 protein, with hypometabolism and atrophy of the anterior temporal lobe (ATL): temporal pole, fusiform gyrus, and inferior temporal gyrus. The pathology is bilateral but asymmetrical, at least in the early stages of the disease, with the left side often more affected. With progression, the atrophy increases on the less-affected side (Brambati et al., 2009) and spreads both posteriorly within the temporal lobes and anteriorly to the orbitofrontal cortex.

SD usually presents as impaired production and comprehension of specific nouns and verbs in the context of fluent and grammatical speech. Asked a question in clinic like “How’s your comprehension?”, a patient with SD might reply “What’s comprehension?” or, more fluently, “You’re going to have to help me here, I don’t know what comprehension is”. The semantic deficit may be most apparent in verbal tasks, which underlies the clinical diagnostic criteria of the semantic variant of primary progressive aphasia (svPPA), a common manifestation of SD. Careful assessment reveals that the impairment also applies to nonverbal abilities, such as choosing two visually presented objects in an array that belong together. It also affects multiple semantic categories (i.e., both living and non-living things and abstract concepts).

This distinctive cross-category, modality-independent semantic deficit, in association with circumscribed atrophy of the ATL, has been a main basis for proposing this region as the hub in the distributed plus hub model. In other words, the hub role of the ATL in semantic memory is suggested by the deficits in semantic memory that arise from specific disruption to this region, consistent with its role in connecting, and collecting information from, the association cortices involved in the representation of components of semantic concepts.

Studying patients with semantic dementia therefore provides a means by which to assess the contribution of the ATL to semantic memory. As well as speaking to the importance of the hub in binding together concepts, studies of the residual semantic knowledge of patients with moderate disease can inform us about the way in which these concepts are coded. For example, it is commonly considered that this region can represent knowledge of specific people, but it remains controversial whether this code is distributed and somewhat redundant, or whether there might be single cells that respond only to certain individuals (Quiroga et al., 2005). While extended neuropsychological assessments can readily show the semantic impairment, there is also much to be learnt from the qualitative observation of patients’ representation of individual concepts, and how these change with disease progression.

The present paper presents drawings of familiar objects, in response to their spoken names, made by patients with SD. With some exceptions (see, e.g., Pozueta et al., 2019), previous studies of drawing in SD have concentrated on a comparison of immediate versus delayed copy of object drawings, where the stimulus was a line drawing of the object (see, e.g., Bozeat et al., 2003; Lambon Ralph & Howard, 2000; Patterson & Erzinçlioğlu, 2008; Patterson et al., 2006). The current study was designed to assess how well SD patients can retrieve and represent identifying features of familiar objects on the basis of the object name alone. Drawing in response to the object’s name avoids potential confounds from representations in visuospatial working memory and/or episodic declarative memory, created by seeing a target picture. Drawing to name measures performance when there is no perceptual connection between the stimulus (object name) and the response (object drawing) other than through semantic knowledge.

This was a partially longitudinal study, which had the primary aim of illustrating the nature of drawings by people with SD, testing the hypotheses that semantic impairment is graded rather than binary, implying a feature-based storage system for semantic knowledge.

## Methods

### Subjects

Drawings were made by 19 patients with diagnoses of semantic dementia (SD), in specialist clinics at Cambridge University Hospitals NHS Trust (Addenbrookes hospital). These patients in fact comprised two cohorts (Table 1)—a contemporary cohort of 12 patients, who attended clinic between 2012 and 2019, for whom same-day standardized assessment with the Addenbrooke's cognitive examination revised edition (ACE-R) was available, and an historical cohort of 7 patients, who conducted their drawings between 1993 and 1999 before the ACE-R existed. This latter cohort undertook the Mini-Mental State Examination (MMSE), Rey Figure copy, and a 64-item naming test that was ultimately published as part of the Cambridge Semantic Memory Test (Adlam et al., 2010). As part of clinical assessment, all patients were asked to produce drawings of living and non-living things in response to the name of the animal or object. Structural T1-weighted MPRAGE 3T MRI scans were acquired within 6 months of the date of the drawings for 11 of the 12 contemporary patients.

**Table 1:** Patient demographic and neuropsychological information

Patient ID	Age	Gender	Atrophy predominance	MMSE (/30)	ACE-R (/100)	ACE-R Naming (/12)
Contemporary Cohort						
1	74	M	Left temporal	27	21	5
2	63	M	Left temporal	26	61	2
3	64	M	Right temporal	27	61	3
4	63	F	Bilateral temporal	19	50	0
5	68	F	Left temporal	24	54	3
6	69	M	Left temporal	20	43	1
7	66	F	Left temporal	28	71	4
8	64	M	Left temporal	26	70	5
9	73	M	Left temporal	27	68	6
10	65	M	Bilateral temporal	25	63	1
11	71	M	Left temporal	15	35	1
12	69	M	Left insula	28	82	11
Historical Cohort						
Patient ID	Age	Gender	Atrophy predominance	MMSE (/30)	Rey Copy (/36)	64-item Naming (/64)
13	66	M	Left temporal	11	26	1
14	61	M	Left temporal	Not available		
15	71	F	Right temporal	26	32	Not available
16	58	F	Left fronto-temporal	22	33	55
17	58	F	Left temporal	24	36	10
18	59	F	Left temporal	8	30	0
19	77	F	Left temporal	18	32	11

### Drawings

The general method of administration was to begin by asking the patient to draw a common animal (e.g., cat, dog). After this, the patient was asked to draw increasingly rarely encountered animals until they consistently responded to the effect of: “I don’t know what that is.” As a result, individual patients did not all draw the same animals. At this stage, patients in the historical cohort were asked to draw inanimate objects (e.g., scissors, envelope, plane), but these items were omitted in the contemporary cohort.

The range of items in our sample is shown in Table 2.

**Table 2:** Range of items assessed in this study

Item	Snodgrass and Vanderwart (1980) typicality score (out of 5)	Number of patient drawings assessed
Domestic animals:		
Dog	4.60	14
Cat	4.22	11
Rabbit	2.95	6
Large animals:		
Horse	3.55	13
Cow	2.42	6
Elephant	2.35	14
Camel	2.08	11
Rhinoceros	1.52	4
Birds:		
Duck	2.75	10
Eagle	2.42	6
Swan	1.97	4
Penguin	1.70	9
Inanimate objects		
Glass	4.78	4
Key	4.85	4

### Scoring drawings

The main focus of this paper is qualitative, with patient produced drawings illustrating the gradual and graded loss of semantic knowledge for rare then common object exemplars in semantic dementia in a more nuanced way than could be achieved by quantitative neuropsychology. However, in supplementary material we present an exploratory quantitative analysis of the drawings, and correlation with neuroimaging.

### Voxel-based morphometry

Scans for the 11 patients scanned in this study were compared against 14 healthy age-matched individuals previously scanned for the study published as Cope et al. (2020). Voxel-based morphometry analysis was performed in SPM12 r6906, running in Matlab2017a. The full analysis pipeline script is available online (https://github.com/thomascope/SD_Drawing/blob/main/SD_full_VBM_script.m). In brief, images were first approximately aligned by coregistration to an average image in Montreal Neurological Institute (MNI) space before segmentation and calculation of total intracranial volume (TIV). After segmentation, a study-specific DARTEL template was created from the 11 patient scans and 11 controls, using default parameters. All subject scans were then warped to this template. The templates were affine aligned to the SPM standard space using ‘Normalise to MNI space’ and the transformation applied to all individual grey-matter segments together with an 8 mm FWHM Gaussian-smoothing kernel.

To assess overall atrophy in the patient group relative to the controls, the resulting images were entered into a full factorial general linear model, with a single factor of group having two levels (patient or control), and age and TIV as covariates of no interest. The model was then estimated in the classical manner, based on restricted maximum likelihood. SPM’s inbuilt method for voxel-wise family-wise error correction was employed (note that this is the most conservative of the available methods for multiple comparison correction, but is appropriate here as the atrophy in semantic dementia is not at all subtle).

For the supplementary information quantitative analysis, single-subject average grey matter density was then extracted using the AAL atlas in six regions of interest: temporal pole, inferior temporal gyrus, and fusiform gyrus, each both left and right. These single subject densities were then correlated with single-subject composite drawing scores, using each of the three normalization methods. Statistical significance was assessed with both Pearson (parametric) and Spearman (nonparametric) methods, using one-sided tests on the basis that volume loss will not conceivably improve performance.

All patients’ individual drawings are collated in a supplementary PDF, labelled so that they can be related to the demographic and neuropsychological data in Table 1. The data and materials for the drawing experiments are therefore available as supplementary material, but we do not have consent to share the neuroimaging data. None of the experiments was preregistered

## Results

### Animal drawings demonstrate a graded dissolution of semantic knowledge

**Fig. 1:**
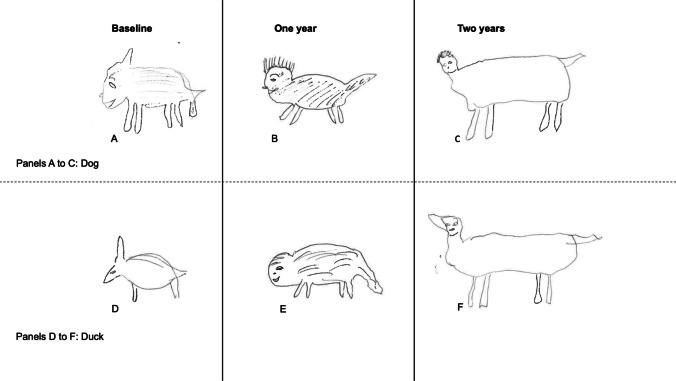
A single SD patient’s drawings of a dog (top row; **A, B, C**) and duck (bottom row; **D, E, F**) over a 4-year timespan. Both animals lose their distinctive features and tend toward the ‘prototypical’ four-legged animal with inappropriate feature intrusions, consistent with a progressive graded dissolution of semantic knowledge

Figure 1 demonstrates two animals, a dog and a duck, drawn by a single patient over a 3-year time span. The basic visual characteristics of both objects remained elicitable by name throughout this time span, implying relative preservation of the animal concepts in this period; however, the specificity of the drawings deteriorated from the earlier stage of disease (baseline) to the latter stage (2 years), suggesting a progressive degeneration of the underlying semantic representation in a graded manner.

The dog, a common ‘template’ animal, preserves its most typical features, but gains a rather human-like face and a less biologically shaped body in late illness. The duck, a moderately familiar animal, initially has a beak, two legs and perhaps a suggestion of wings, but from one year onwards demonstrates intrusion in the form of the four legs and square-ish body, more characteristic of highly familiar animals.

**Fig. 2:**
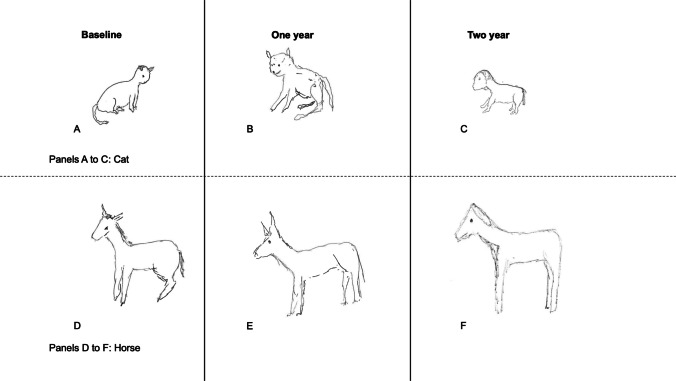
A single SD patient’s drawing of a cat (top row) and horse (bottom row) across a 2-year time-frame. At initial assessment (baseline), the horse has a distinct mane, upturned ears, good hind leg muscle bulk, and a hairy, bushy tail (**D**), but by 1 year, it has lost its mane, and some hind leg muscle bulk, and the tail is now a single line (**E**). At baseline, the cat is drawn sitting in a typical feline posture (**A**), but by 1 year, its general body proportions have distorted, with a loss of the curvature of its spine, and hind leg muscle bulk. A smile is drawn on its face, representing the intrusion of features typically found in humans (**B**). By 2 years, both the horse (**F**) and cat (**C**) are significantly different from baseline and have lost several of the distinctive features highlighted above. They now share a resemblance with one another in their orientation (facing left), posture (upright, with four distinct straight legs) and proportions. The horse retains an elongated neck and long thin legs, but has lost its mane, its upturned ears, its tail and hind leg muscle bulk (**F**)

Figure 2 demonstrates a similar degeneration of semantic information for a cat and a horse, drawn by a different patient over a 2-year time frame. Here, progressive feature loss is noticeable. Drawings in the earliest stage of disease captured in this patient show a recognizably distinct cat and horse, which become less recognizable later in disease course. Towards the end, the two become more similar to one another in their proportions and general appearance, though they retain a difference in size; we have noted in the drawing and indeed other tasks, such as verbal definitions (e.g., asking the patient to explain what an elephant is), that the size of animals is one of the more robust aspects of semantic knowledge as SD progresses. Of note, this patient’s drawings display a progressive deterioration in the distinctiveness of the individual features that comprise the target animals. For example, by 2 years post-baseline, both the cat and the horse have lost their characteristic stand-up ears; the horse’s tail first loses volume and then disappears altogether. These feature changes indicate a progressive loss in the amount of visual semantic information with disease progression, rather than a sudden and complete loss of the entire concept of each animal.

**Fig. 3:**
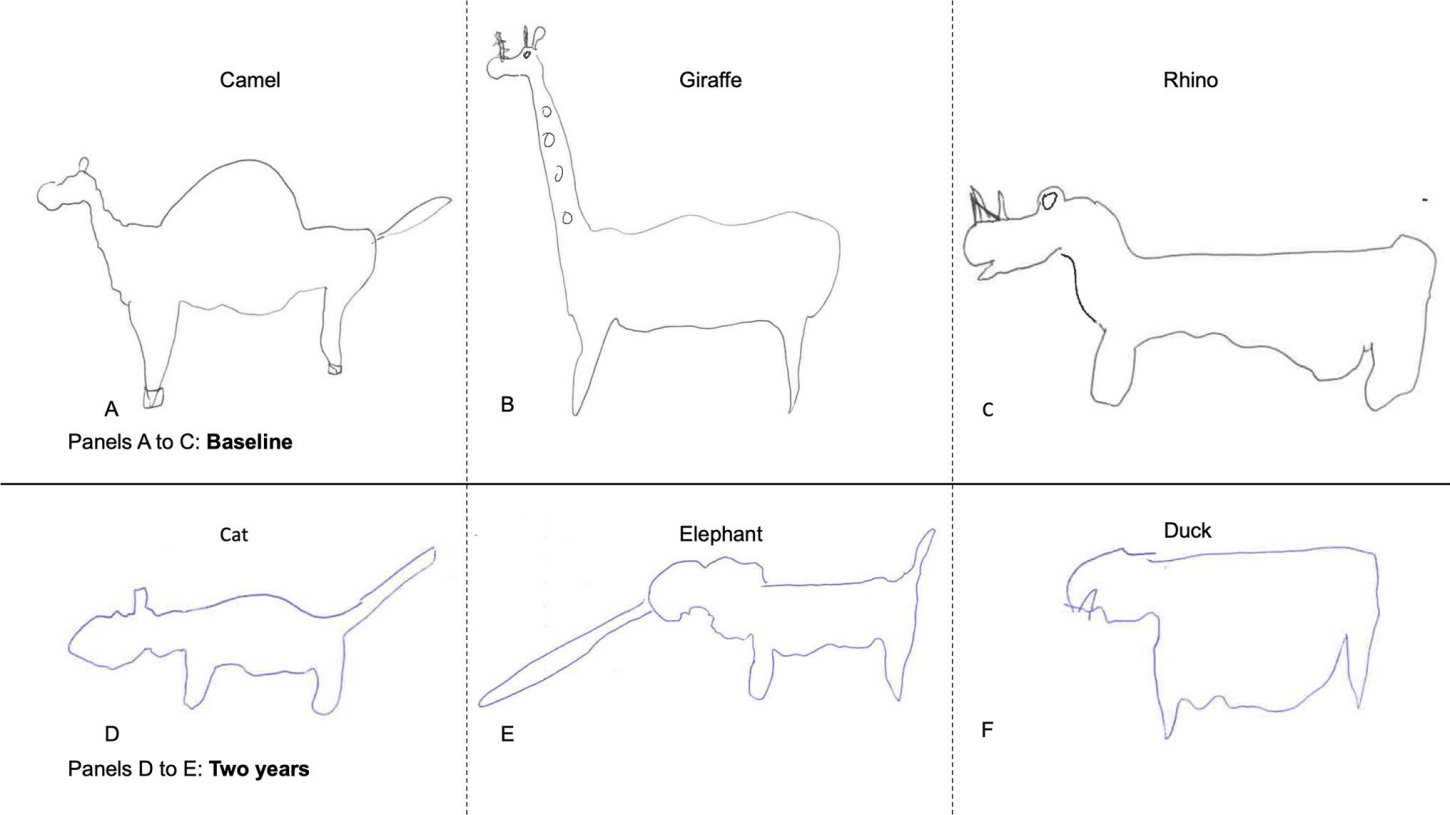
A single SD patient’s drawings of a camel (**A**), giraffe (**B**) and rhino (**C**) at initial assessment (baseline). At this time, all three animals are distinct and resemble their living counterparts; the camel has a hump; the giraffe has a tall neck, spots, and misplaced but present horns; and the rhino has a stocky body and large head, with two horns. Two years later, the same patient draws a cat (**D**), elephant (**E**), and duck (**F**), which at this stage no longer obviously resemble their living counterparts and are much less distinct from one another, with the duck resembling a land animal/mammal, exemplifying the ‘prototypicality effect’ that accompanies late-stage semantic dementia

In more clinically advanced disease, the ‘prototypicality effect’, whereby semantic representations degrade to their most basic, most generalizable form, becomes apparent. Figure 3A–C shows three uncommon animals drawn by a third patient at a single time-point (baseline); while degraded, the drawings are somewhat representative of their living counterparts. The same patient, 2 years later, draws three different animals, two of which are relatively common (cat and duck) and one of which is uncommon (elephant) in the UK. All three drawings here share a similar template or ‘prototypical’ structure, with a head, body, and two legs. The elephant retains a trunk as its sole distinctive feature; the duck has lost all features that would identify it as a bird and is drawn to resemble a land animal. Semantic knowledge of these animals in this patient has degraded over time to such an extent that his drawings no longer distinguish a bird from a mammal. This is consistent with the progressive degeneration of a neural network in which connection strength is based on the frequency/familiarity of concepts; the most common and earliest encountered animals for our patient demographic would likely be domestic, and the most common domestic animals (dogs and cats) would be mammalian.

### Semantic impairment is independent of the concept category in semantic dementia

**Fig. 4:**
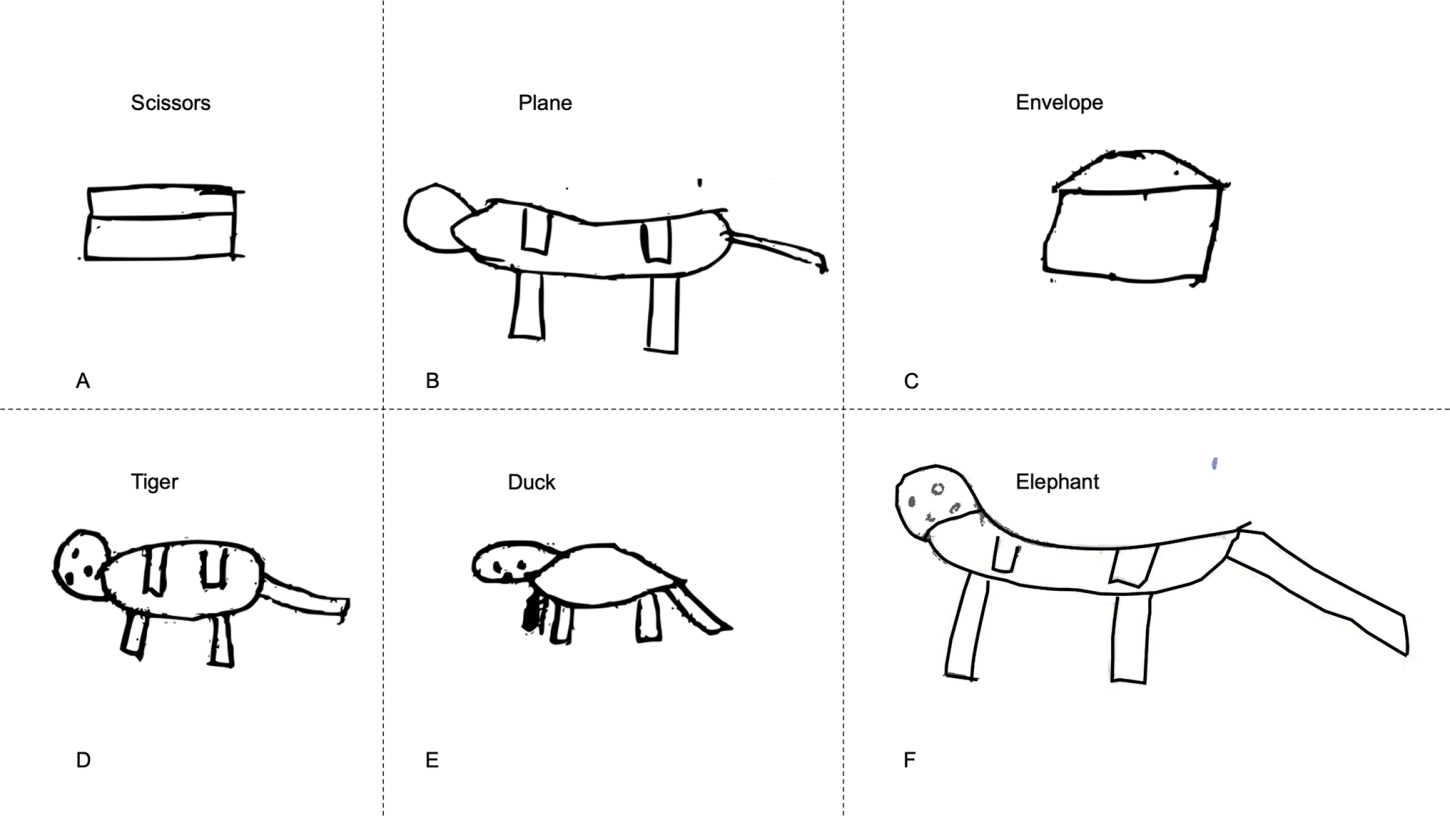
Inanimate objects become more box-like in their appearance, especially the scissors (**A**). The plane (**B**) shows the intrusion of animal features in the form of four wings—drawn identically to the way the patient has drawn legs in the tiger (**D**) and duck (**E**)—rather than two, and a tail, but maintains an elongated body (perhaps representing the fuselage). It develops a ‘head’ without facial characteristics. The tiger, duck, and elephant share resemblance with a similar body and head structure and each with four legs

Semantic impairments in SD are not confined to a single category, and are present for both animate and inanimate objects (Fig. 4). Inanimate objects become more box-like in their representations (Fig. 4A), and animals become less distinguishable from each other (Fig. 4B). In fact, sometimes even the animate/inanimate distinction becomes blurred: apart from the facial features, the plane and the tiger look almost identical.

### Graded dissolution of semantic knowledge is the norm across the SD disease spectrum

In the Supplementary Materials, we provide example drawings from all 19 patients. This shows that graded dissolution of semantic knowledge is the norm across the SD disease spectrum, underscoring our claim that picture drawing from name is a useful test contributing to the clinical diagnosis of SD. A similar conclusion has been offered by Pozueta et al. (2019).

### Atrophy of the left inferior temporal gyrus underlies impaired semantics

**Fig. 5:**
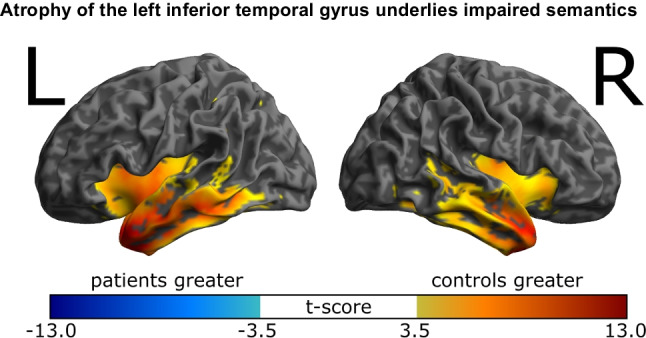
Voxel-based morphometry of 11 patients with SD against 14 healthy age-matched controls, thresholded at FWE *p* < .05. (Colour figure online)

Voxel-based morphometry (Fig. 5) demonstrated the expected pattern of atrophy in SD, with bilateral temporal lobe grey matter loss, more severe and extensive on the left, [−42 4 −23] peak, *t*(22) = 13.0, *p* < .001, cluster volume 51,948 voxels, than the right, [38 14 −29] peak, *t*(22) = 10.6, *p* < .001, cluster volume 24,102 voxels, involving insula, and reducing in severity with posterior distance from the temporal pole.

In the Supplementary Materials we present a preliminary quantitative analysis that suggests animal drawing performance may positively correlate with grey matter volume in the left inferior temporal gyrus.

## Discussion

Line drawings of objects in response to their names provide insights into the graded semantic deficit that results from neurodegeneration of the anterior temporal lobe. Our study makes two key contributions.

First, the patients’ drawings demonstrate that the impairment in SD is independent of the modality of both the input to and output from semantic memory. It has been amply established that these patients gradually lose the ability to name real (or pictures of) objects and animals. If tested longitudinally, object naming displays precisely the kind of progressive loss of specificity observed in our drawing study. For example, tested four times over 1.5 years, an SD patient named a picture of an elephant as (1) elephant, (2) horse, (3) horse, (4) animal, and a chicken as (1) chicken, (2) chicken, (3) bird, (4) animal (Hodges et al., 1995). The drawing to name task in this study is exactly the opposite of the more common test of naming to object, in the modality of both the stimulus and the response; yet the nature of the deterioration over time is identical in the two tasks. Both tasks lack any surface-level clue to the response, which can only be based on the semantic information activated by the picture for naming or the name for drawing. This central semantic deficit is observed in a range of categories, distinguished at their most fundamental level into inanimate and animate objects (Fig. 4).

Second, the drawing-to-name task reveals that the degradation of conceptual knowledge in SD is graded by both the familiarity and the typicality of the target object, as previously demonstrated for object naming (Woollams et al., 2008). Specifically, when patients with SD are asked to draw a named object, there are three possible outcomes: (1) at one extreme, they may produce a recognizable and reasonably comprehensive drawing; (2) at the other extreme, they may say: “I don’t know what that is”; (3) in between, and most tellingly, they may produce an imperfect approximation retaining some but not all of the characteristic features. In other words, they only produce a subset of the semantic attributes that together constitute a whole concept. Often, this third outcome is seen most clearly for animals of moderate familiarity and typicality. As in previous delayed-copy object drawing studies (e.g., Bozeat et al., 2003), the production of typical features, such as the four legs in an animal, is observed in the greatest number of patients cross-sectionally, and for the longest duration longitudinally (Figs. 1, 2 and 3). Indeed, typical features such as the fairly flat ‘human-like’ faces and backs of most mammals replace distinctive features that are limited to one or very few exemplars of a category of object, such as the trunk extending from an elephant’s face or the hump(s) protruding from a camel’s back. This transition from specific to generic occurs gradually over months and years, as ATL atrophy becomes more severe, and progresses further back along the inferior temporal lobe (Fig. 5).

These two results highlight the widespread disruption to semantic memory that is apparent in SD patients. Since in all but the very late stages of SD, neuropathology is limited to the ATL (Bang et al., 2015), these results are consistent with the central role of this region within the neural systems underpinning semantic memory. In a distributed-only model of semantic memory, there is no region of cortex which, when dysfunctional or atrophied, leads to a transmodal and pan-category semantic impairment (Patterson et al., 2007). In other words, the widespread semantic deficits characteristic of SD patients, who have circumscribed ATL atrophy, are consistent with a distributed plus hub model, and suggestive of an anterior and inferior temporal basis for the hub. Note that all of our patients had bilateral atrophy, as is the norm in SD; the capacity of the cross-hemispheric ATL to compensate for unilateral loss remains an open research area (Kocsis et al., 2023; Rice et al., 2018).

The mechanism by which objects are coded in the ventral visual stream is controversial (Reddy & Kanwisher, 2006). Single-electrode mapping studies in humans have detected neurons that show object selectivity independent of object size and viewing angle in the medial temporal lobe (Quiroga et al., 2005), consistent with an invariant coding hypothesis. Taken to the extreme, a single neuron may be thought to be responsible for representing a concept such as a specific individual (Quiroga et al., 2008). Conversely, nonhuman primate single-unit recording studies have shown that neurons in the inferior temporal cortex, considered to represent the apex of the ventral visual stream, can be trained to recognize different and overlapping aspects of a previously unfamiliar object, in a manner consistent with a population code model of visual object memory (Logothetis et al., 1995). In our study, three patients produced drawings at three different time points in their disease course, allowing a qualitative analysis of the temporal degradation of semantic knowledge with disease progression (and ATL atrophy). Here, although individual concepts became less recognizable and showed phenomena such as feature intrusion, some version of the concept remained partially accessible throughout the disease course. In addition, the individual features of objects drawn by these patients demonstrated graded degradation, such as the horse’s tail in Fig. 2. To ascertain directly how this progressive, part-based loss of semantic attributes relates to neural representations will require physiological recordings, either noninvasively (e.g., Cope et al., 2023) or with invasive recordings (e.g., Suthana & Fried, 2012).

Many previous studies have explored the semantic deficits observed in patients with SD, using a variety of stimulus and output modalities, often in the format of picture recognition or naming (Lambon Ralph et al., 1999; Rogers et al., 2003). Bozeat et al. (2003) examined a cross-sectional cohort of SD patients, using both drawing-to-name and delayed-copy drawing (where patients are shown a picture of an object and, ~10 seconds after its removal, are asked to draw what they have just seen), to assess degradation in semantic knowledge. In the present study, several of these findings are independently reproduced using the object-drawing-to-name paradigm. The present paradigm avoids the potentially confounding effects of visuospatial working memory encountered in delayed-copy drawing experiments and allows an assessment of conceptual knowledge in a format where there is no perceptual connection between the stimulus and response. In addition, for three of our patients, a longitudinal analysis of drawings produced over time exemplified the progressive degradation in semantic knowledge observed in SD.

Although the initial presentation of SD is characteristic amongst the frontotemporal lobar degeneration syndromes (Bang et al., 2015), we retrospectively analyzed anterior temporal lobe atrophy in the subset of patients for whom we had MRI-brain imaging. This primarily serves to validate the clinical diagnoses of patients, and to identify the neuroanatomical location of their temporal lobe lesions. MRI confirmed the expected atrophy of the anterior temporal lobes, consistent with previous work (Gorno-Tempini et al., 2004; Mummery et al., 1999). The quantitative analyses that we present in the Supplementary Information, correlating regional atrophy with drawing performance, should be viewed as initial and exploratory. Scoring drawings inevitably has an element of subjectivity, and these rare patients present to clinic at different stages of their illness, meaning that not all were capable of producing drawings of the same objects, and a form of ratio normalization was required such that producing a good drawing of a rare object carried more weight than for a common one. Finally, the small sample size means that our correlation analyses are underpowered to detect small and medium effect sizes, meaning that the analyses are necessarily coarse-grained. However, our finding that animal drawing performance may positively correlate with grey matter volume in the left inferior temporal gyrus is in keeping with previous demonstrations of the importance of this region for semantic processing (Shimotake et al., 2015).

Overall, we propose that line drawings of animals and objects provide insights into the transmodal semantic deficit in SD. Our results are consistent with a distributed-plus-hub model of semantic memory. The graded nature of the deficit in semantic performance observed in our subset of longitudinally observed patients suggests that the temporal lobe binds part-based semantic attributes in its central convergence zone. The primary results in this paper are qualitative, with Figs. 1, 2, 3 and 4 demonstrating that patients showed a continuum of conceptual knowledge loss rather than a binary ‘present’ or ‘absent’ state.

## Supplementary information

Below is the link to the electronic supplementary material.

Supplementary file1 (PDF 4040 KB)

Supplementary file2 (DOCX 1019 KB)

## Abbreviations

SD: semantic dementia
ATL: anterior temporal lobe
