# Supplementary material for: Drawing from name in semantic dementia reveals graded object knowledge representations in anterior temporal lobe
Source: Mem Cognit. 2024 May 22;53(1):428–37. doi: 10.3758/s13421-024-01578-9 (PMC11779775; doi:10.3758/s13421-024-01578-9)
Supplement: Supplementary file 1 — Supplementary file1 (PDF 4040 KB) [file 13421_2024_1578_MOESM1_ESM.pdf]

Supplementary figures

Patient 1

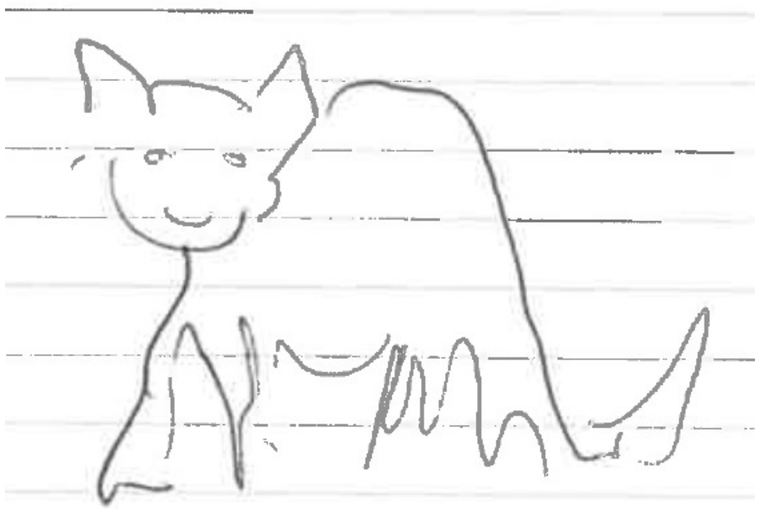

Cat

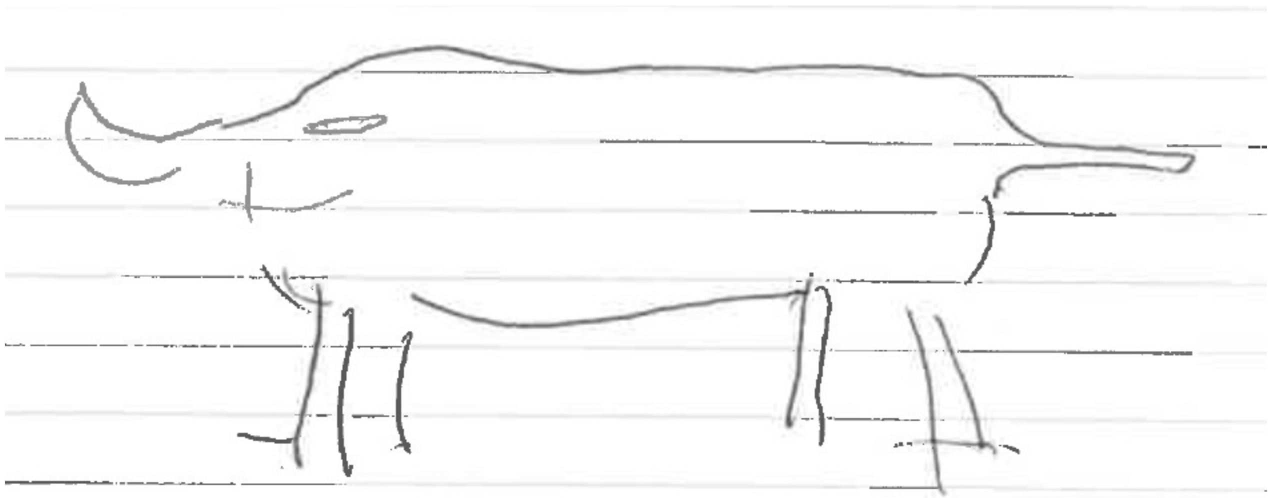

Rhino

Patient 2 (baseline)

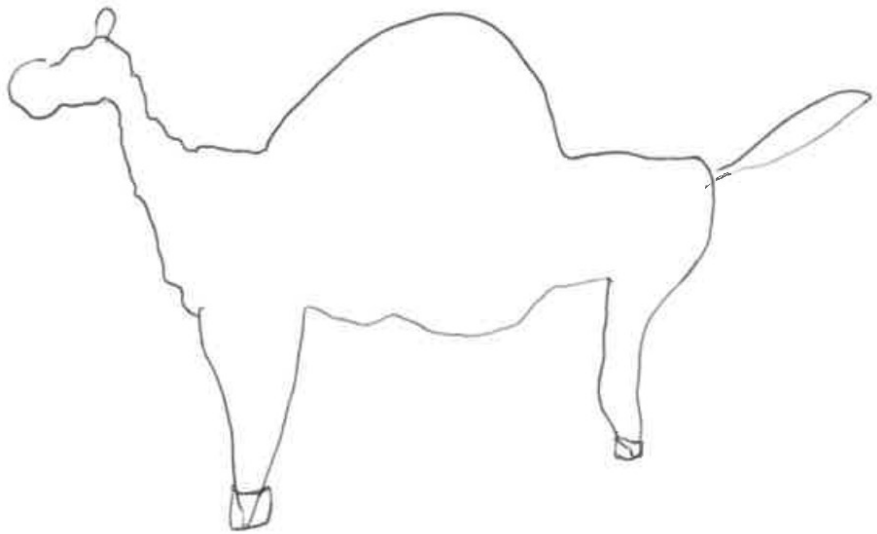

Camel

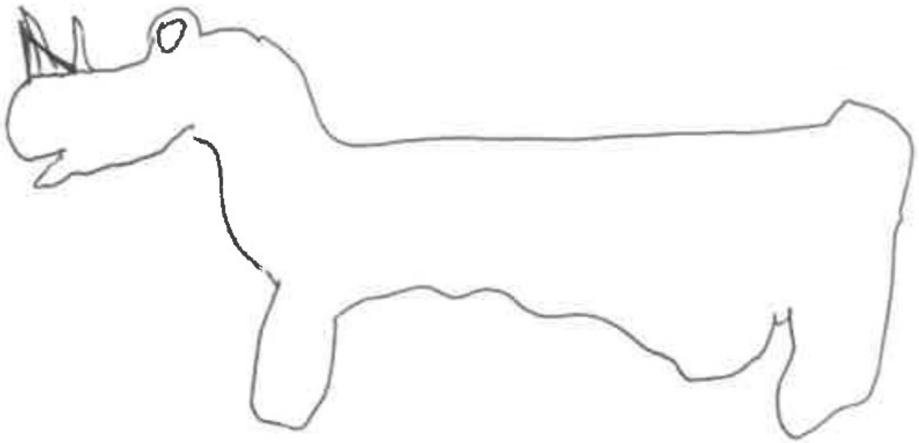

Rhino

Patient 2 (two years)

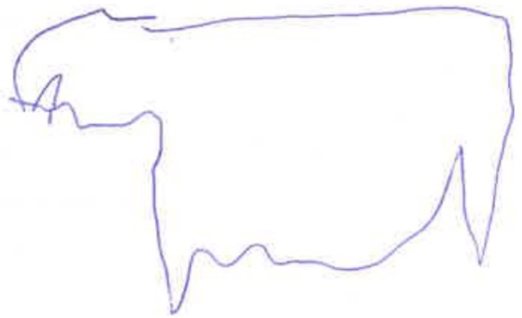

Duck

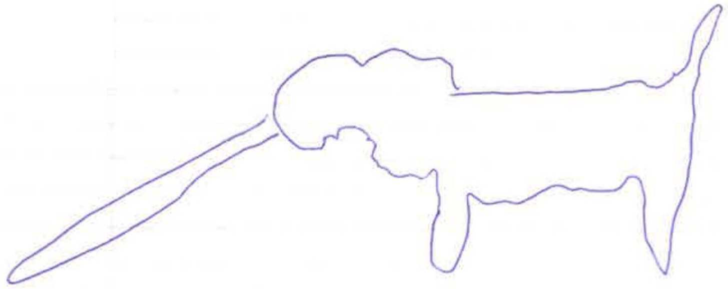

Elephant

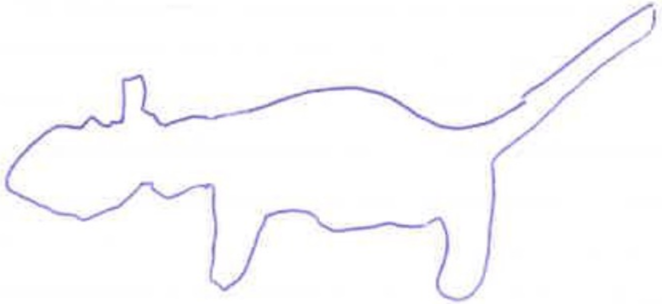

Cat

Patient 3

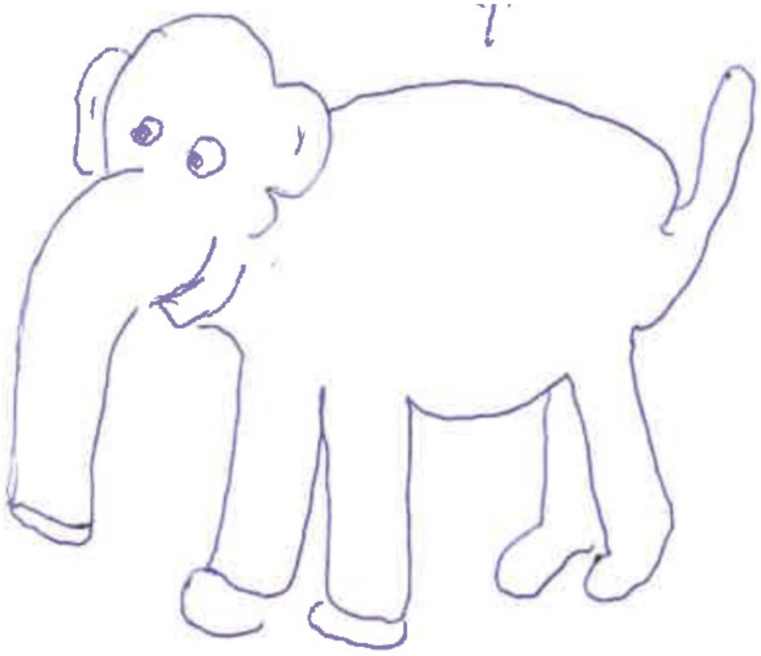

Elephant

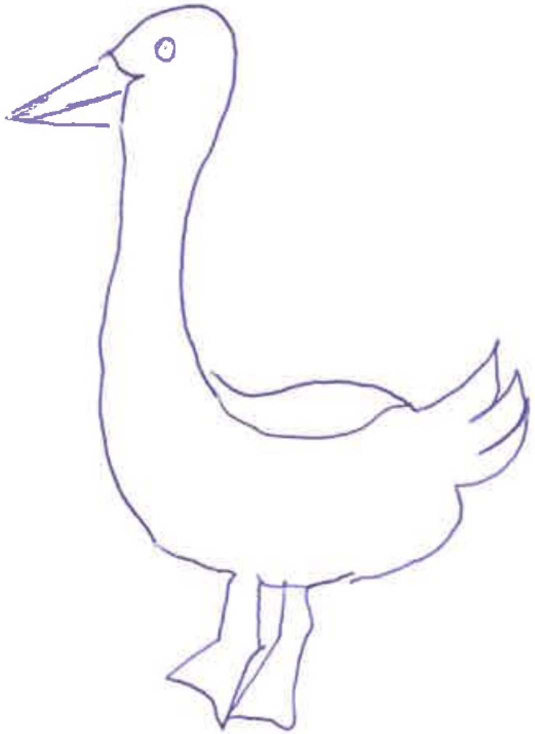

Swan

Patient 4 (baseline)

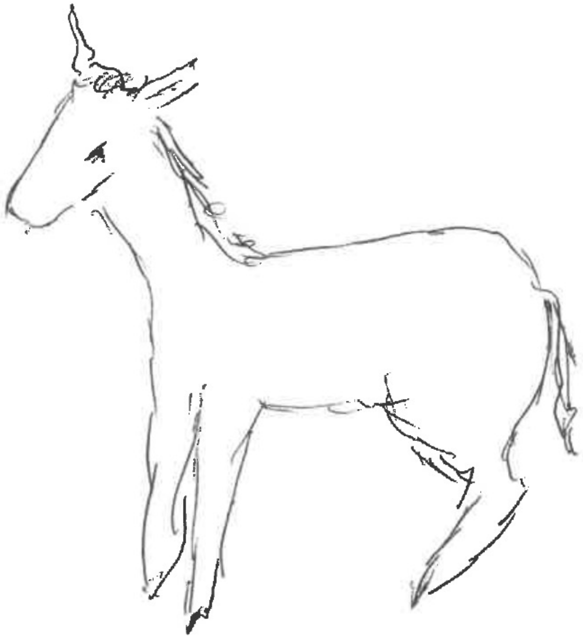

Horse

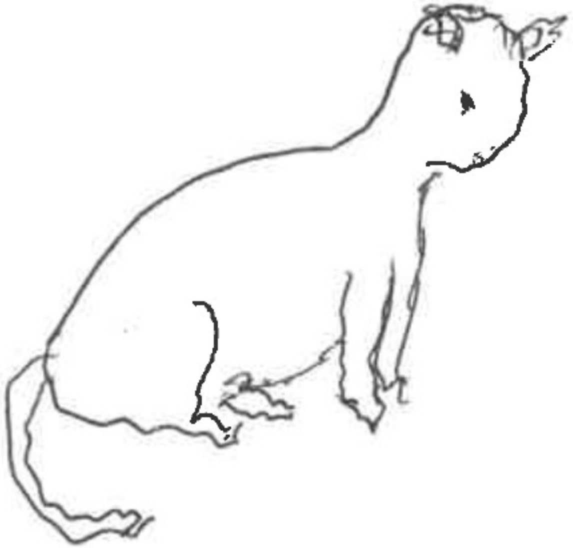

Cat

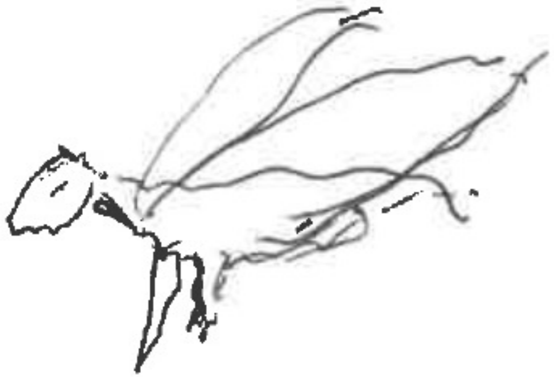

Duck

Patient 4 (one year)

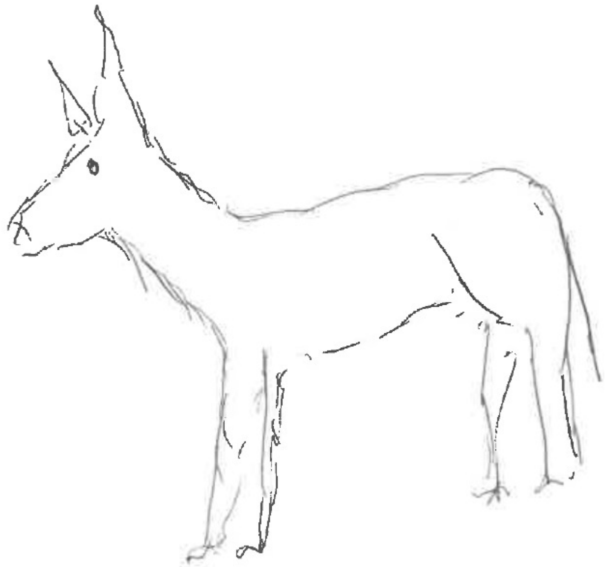

Horse

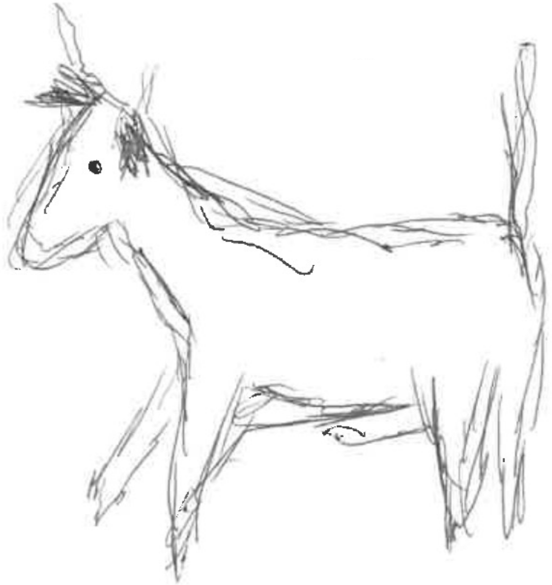

Dog

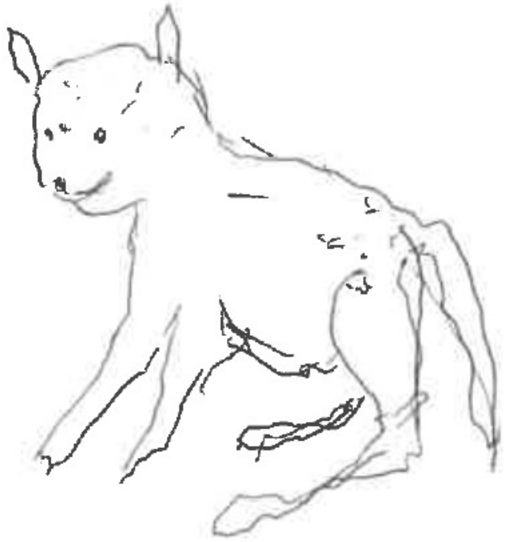

Cat

Patient 4 (two years)

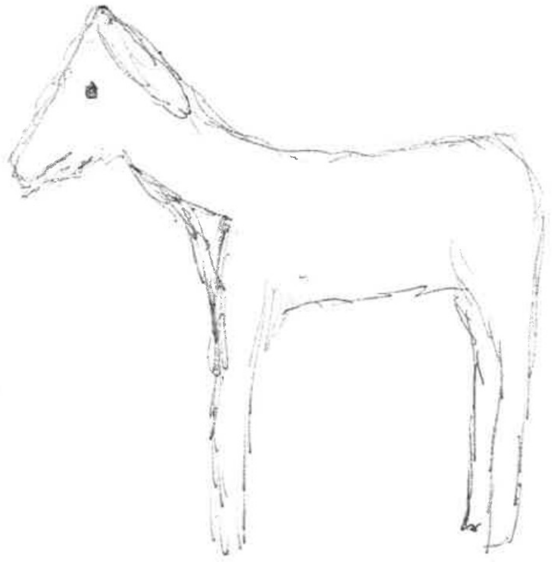

Horse

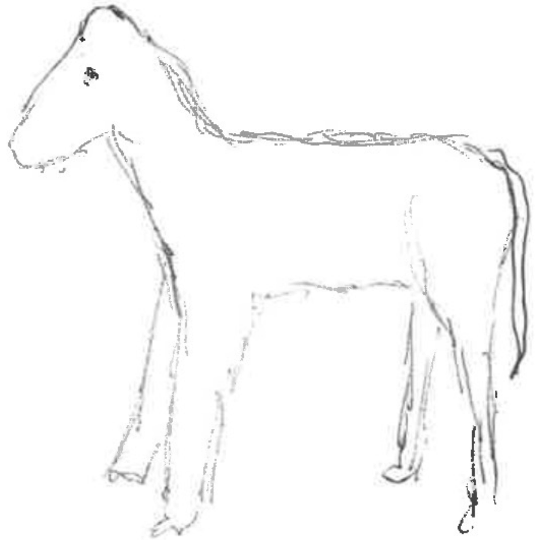

Dog

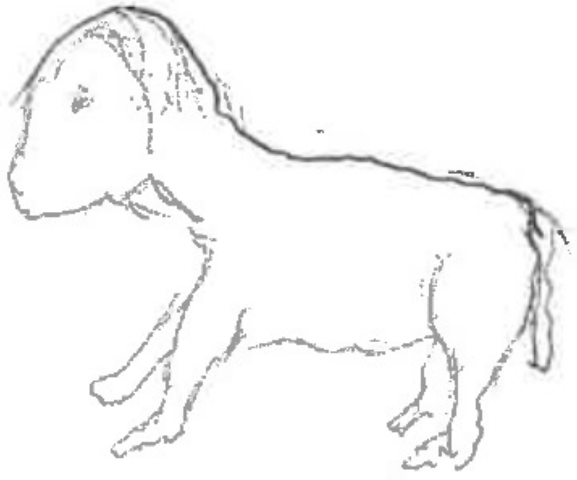

Cat

Patient 5 (baseline)

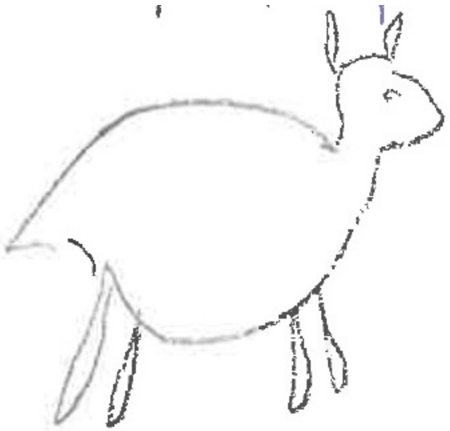

Elephant

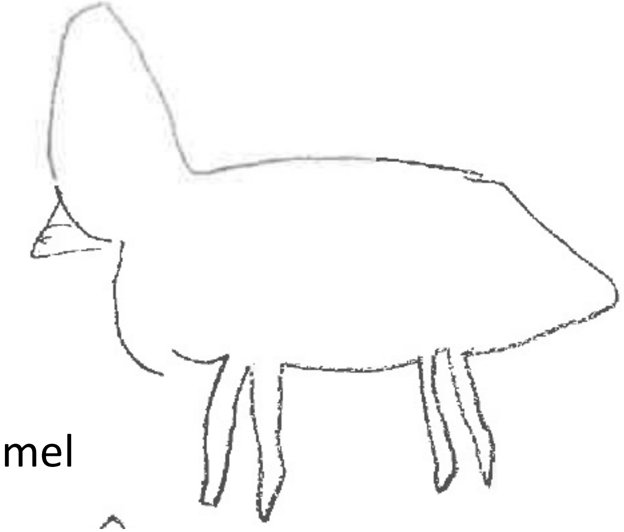

Camel

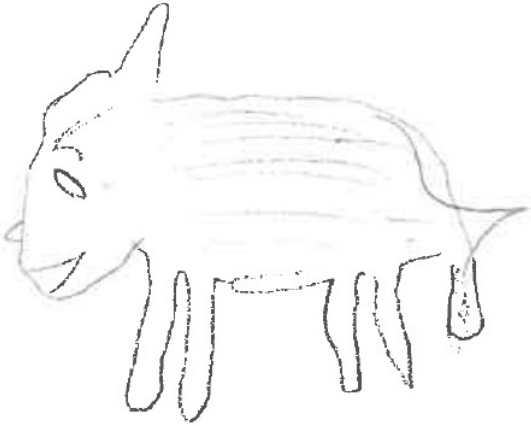

Dog

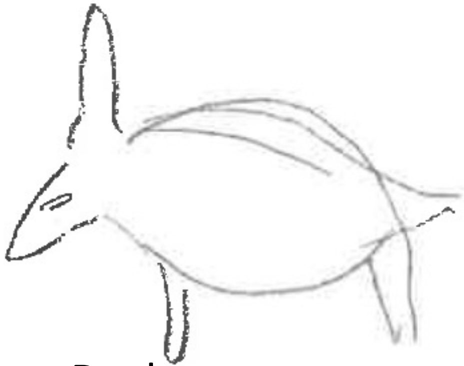

Duck

Patient 5 (7 months)

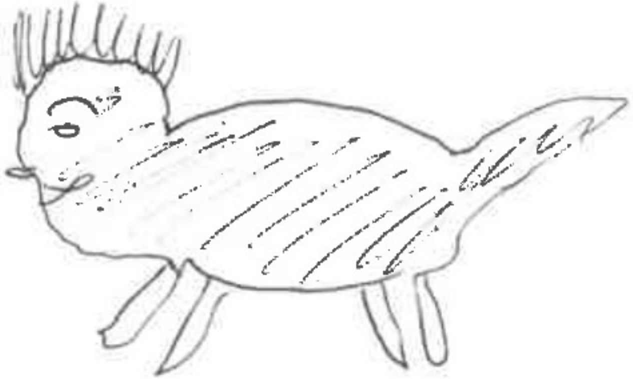

Dog

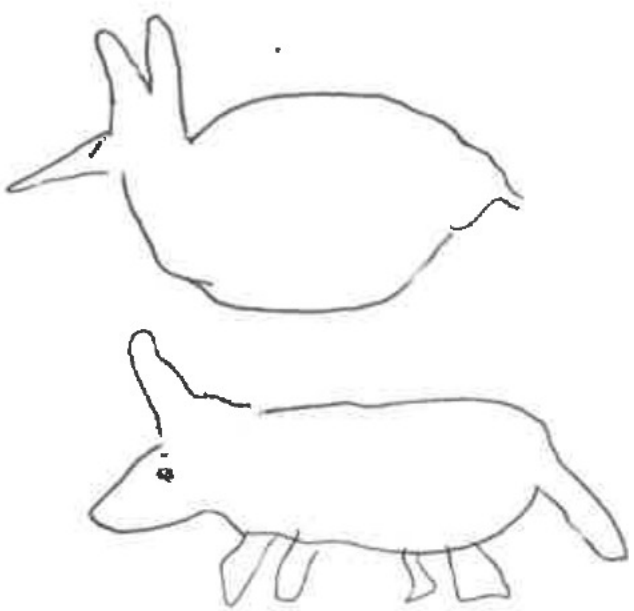

Elephant

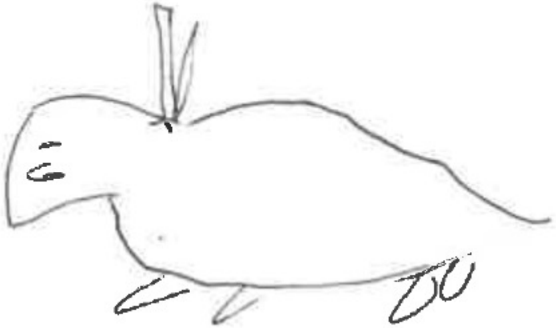

Duck

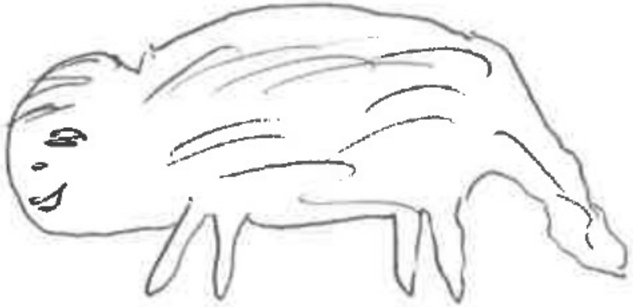

Cat

Patient 6

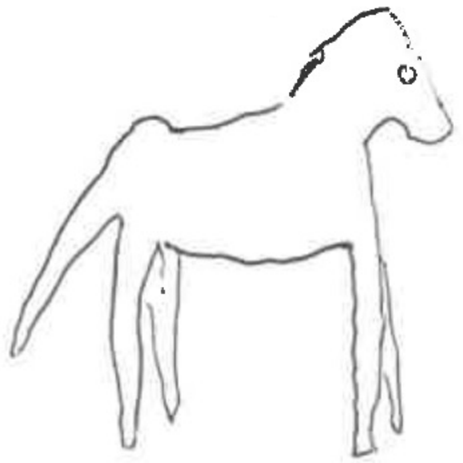

Dog

Patient 7 (baseline)

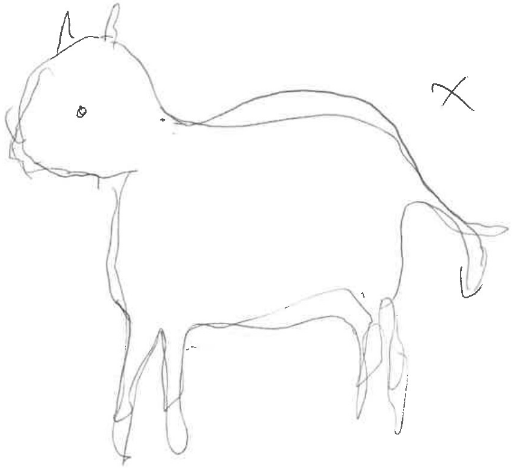

Elephant

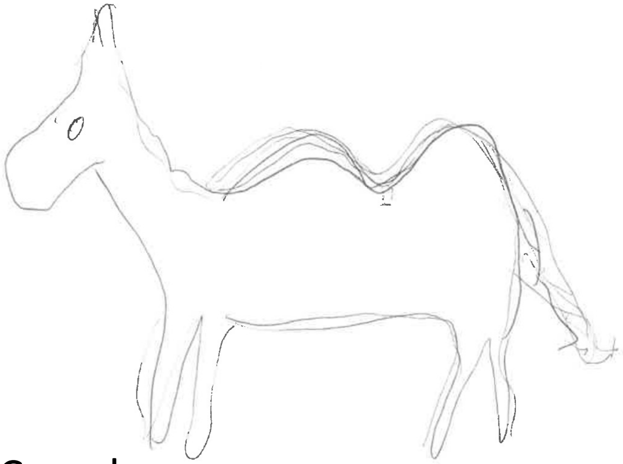

Camel

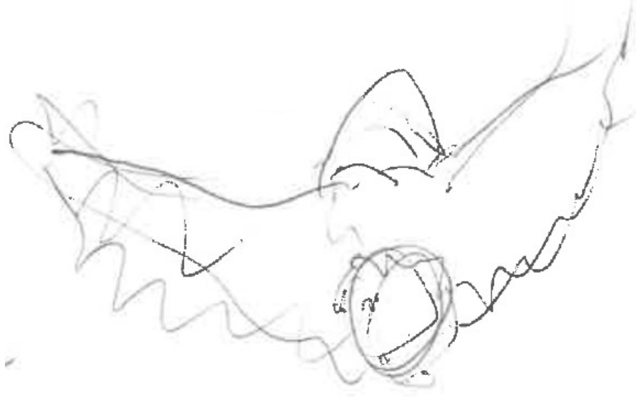

Eagle

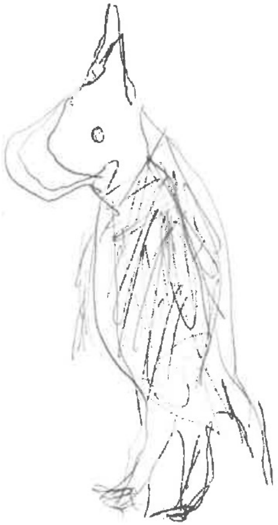

Penguin

Patient 7 (5 months)

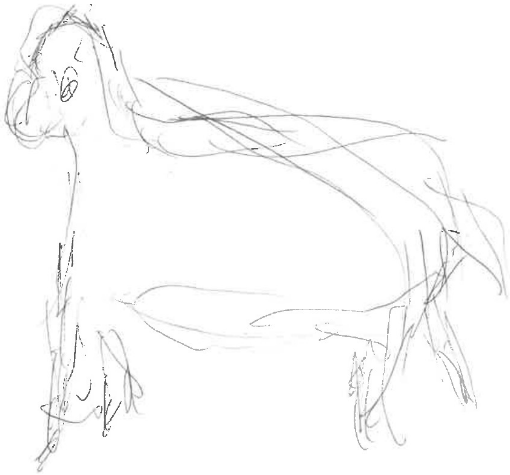

Elephant

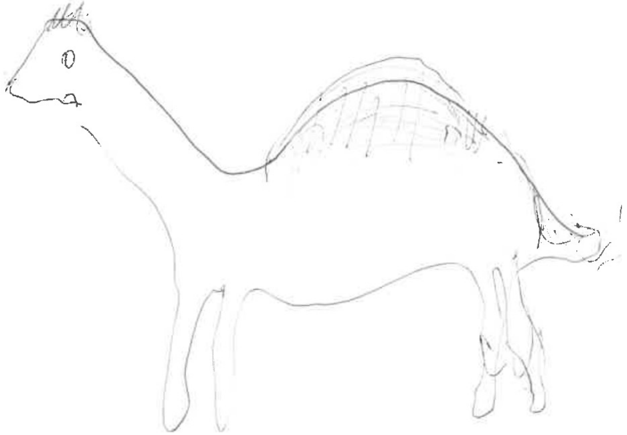

Camel

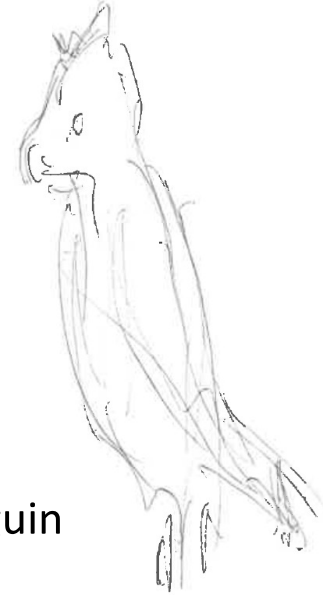

Penguin

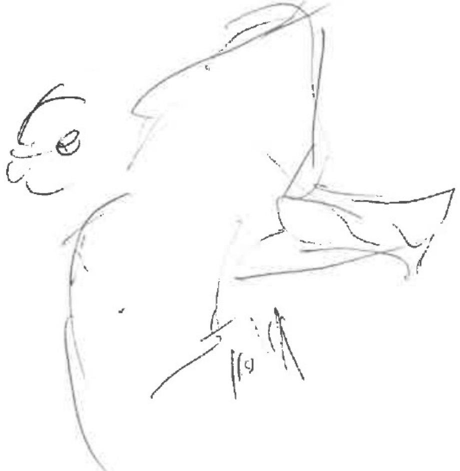

Eagle

Patient 8 (baseline)

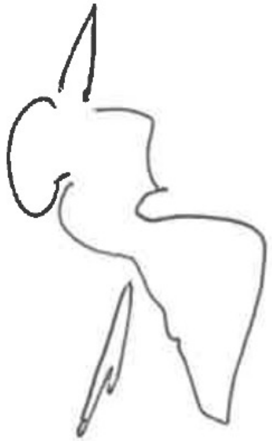

Duck

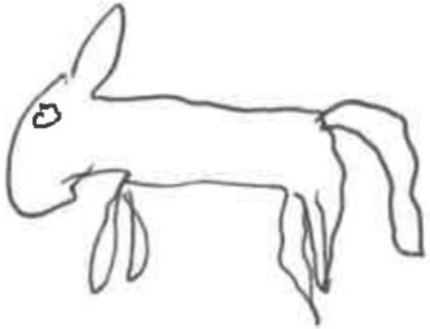

Rabbit

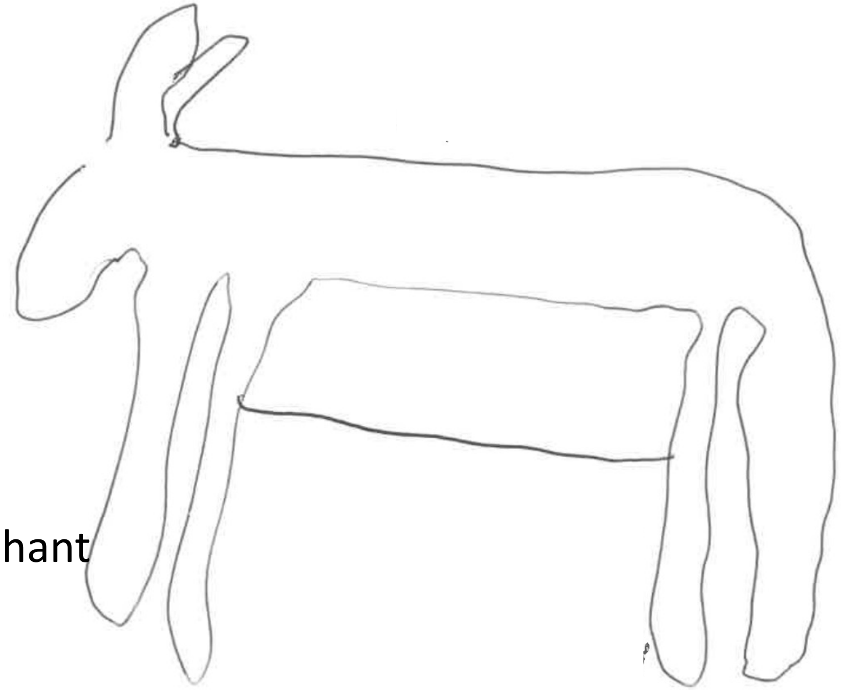

Elephant

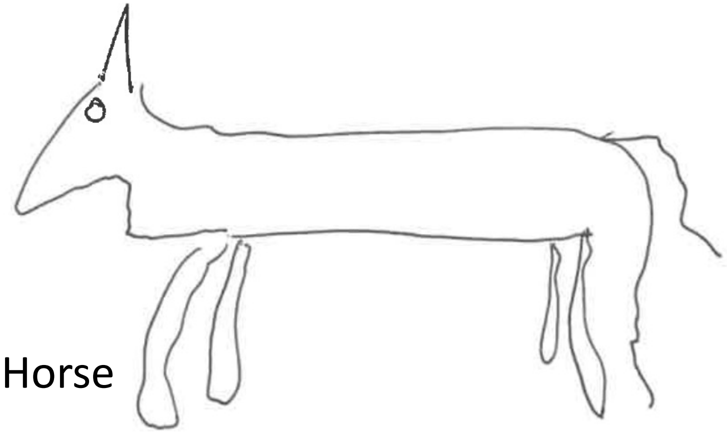

Horse

Patient 9

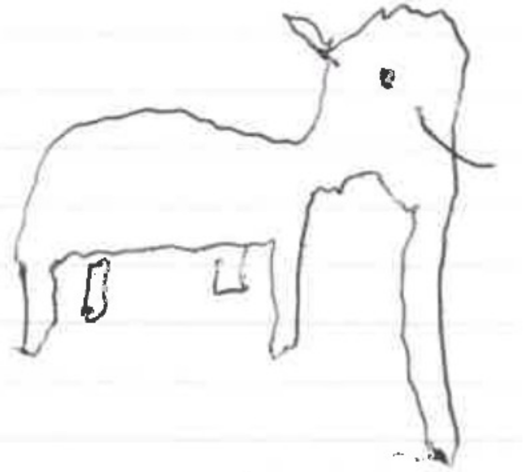

Elephant

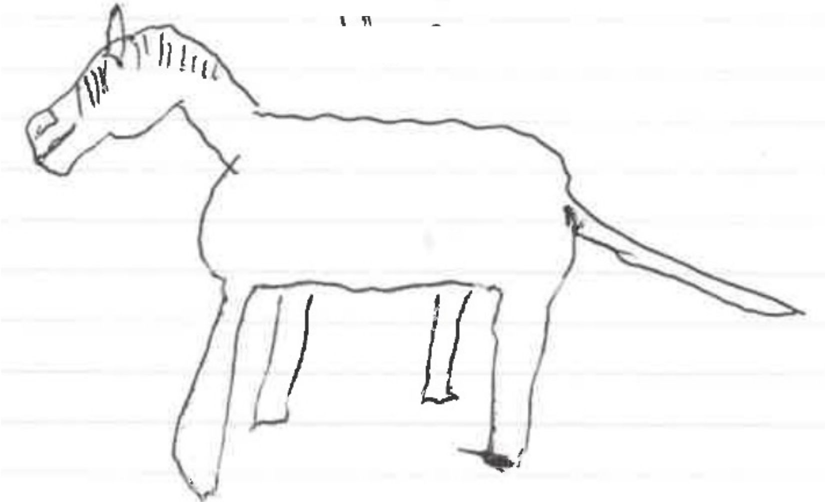

Horse

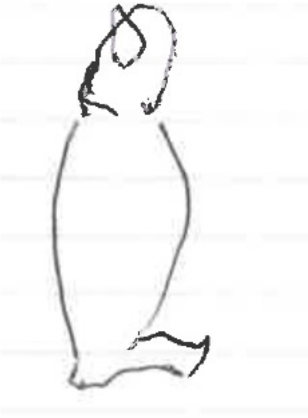

Penguin

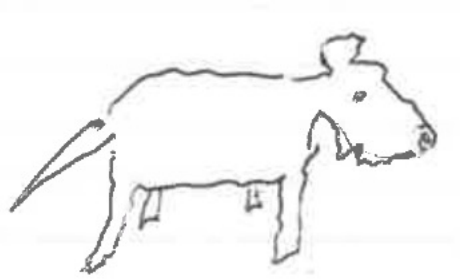

Dog

Patient 10

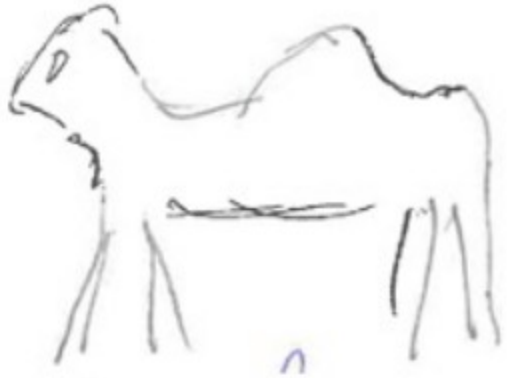

Camel

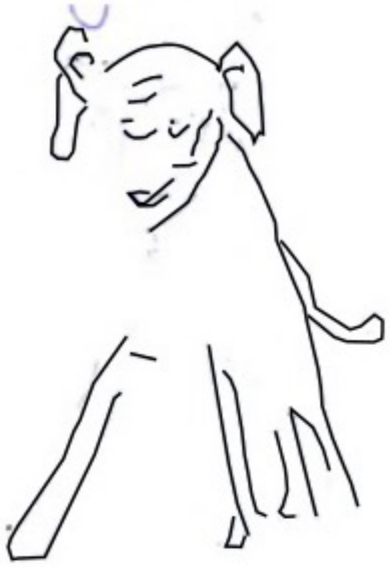

Dog

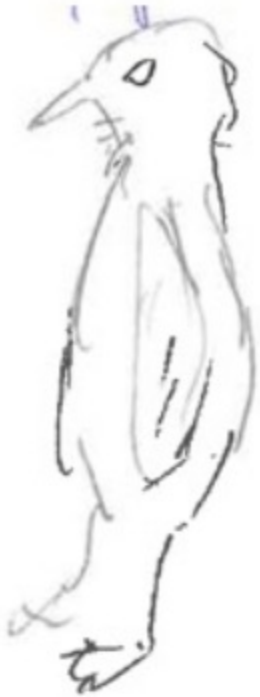

Penguin

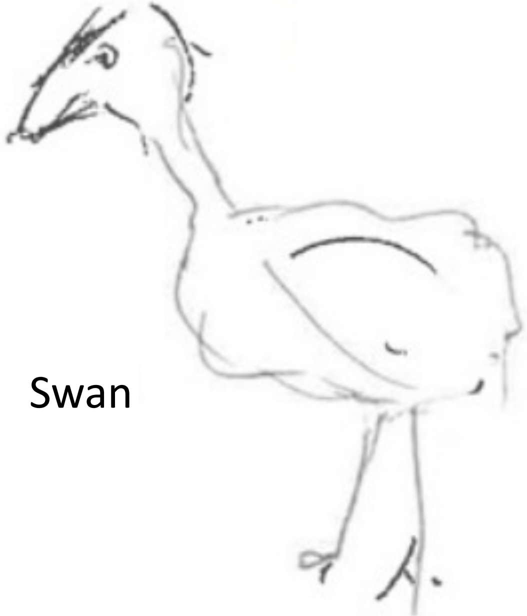

Swan

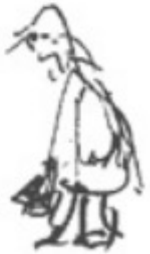

Duck

Patient 11

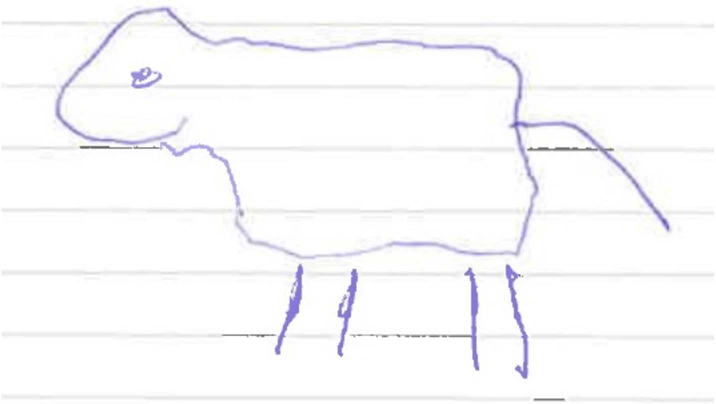

Cow

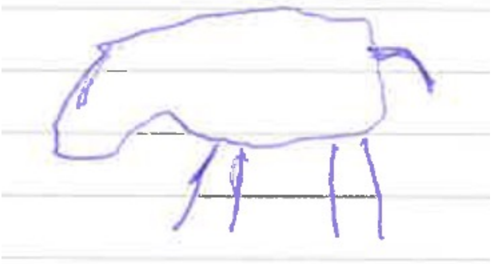

Dog

Patient 12

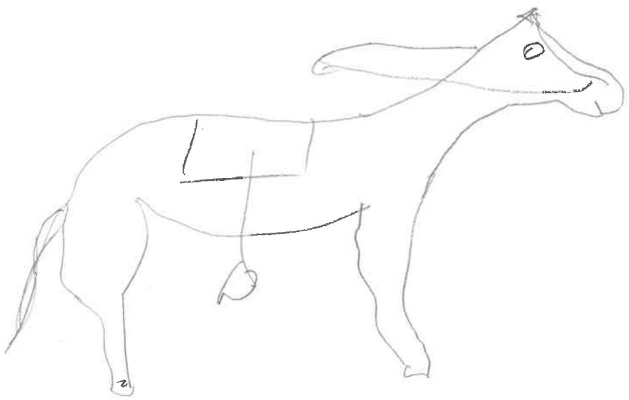

Horse

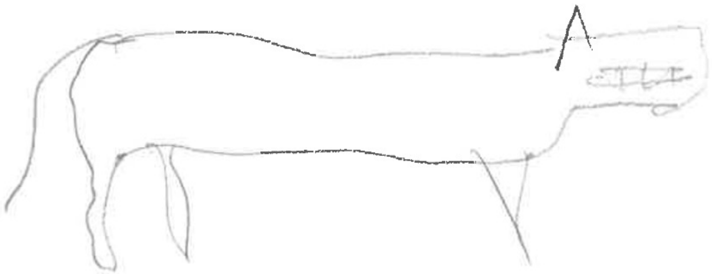

Dog

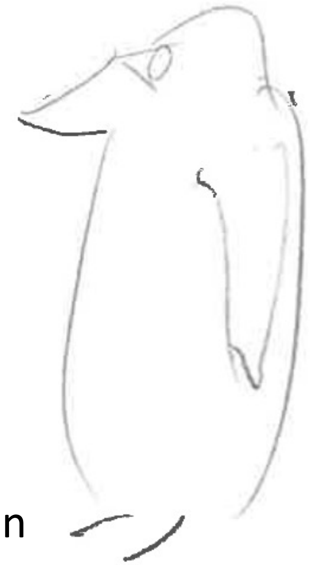

Penguin

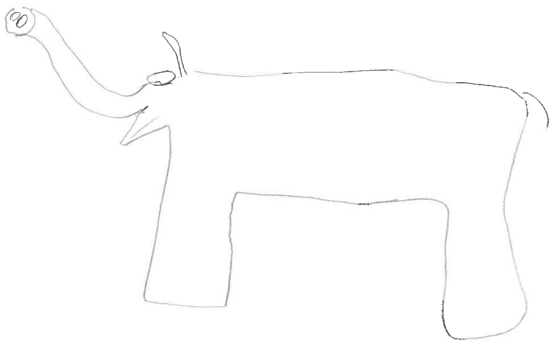

Elephant

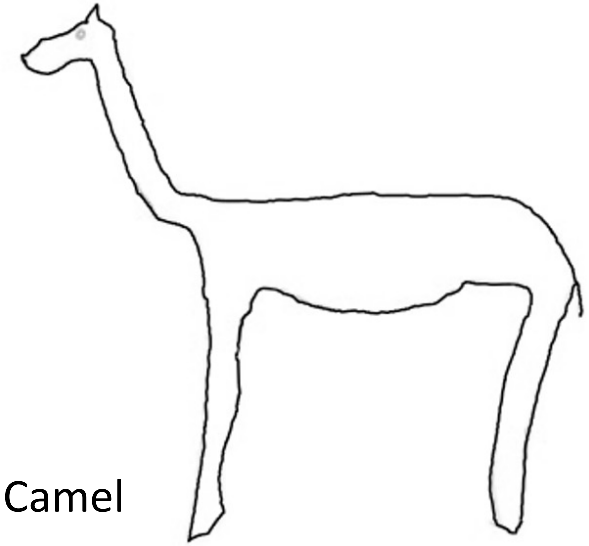

Camel

Patient 13 (baseline)

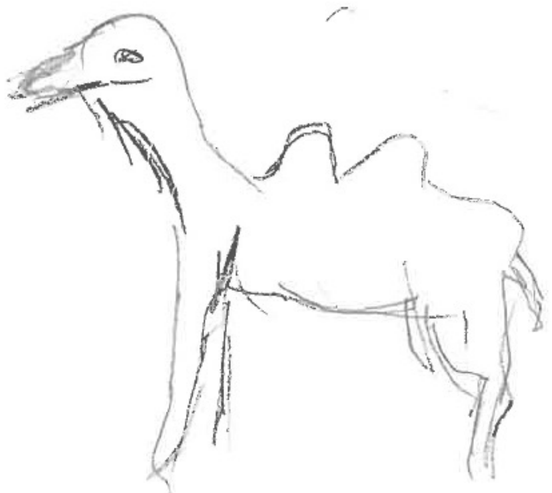
Camel

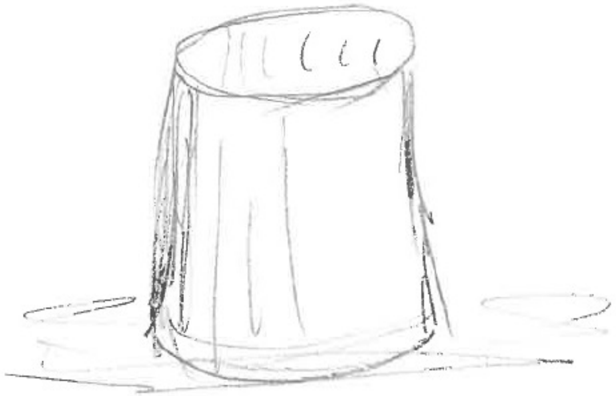
Glass

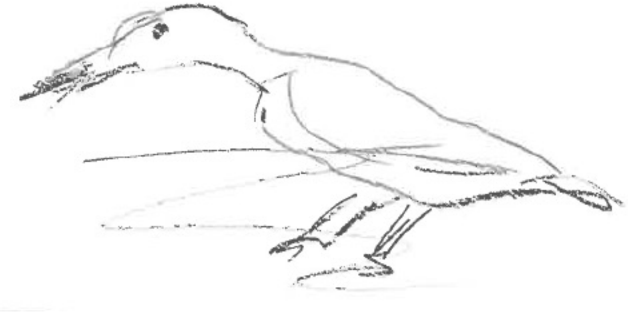
Duck

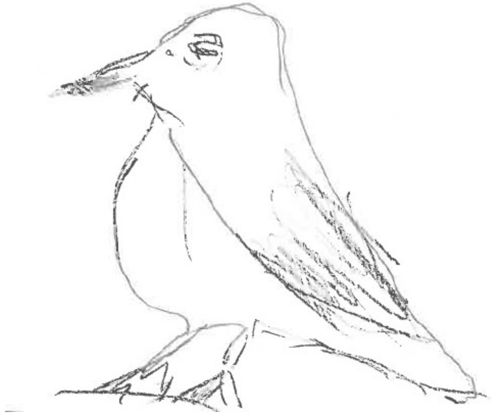
Eagle

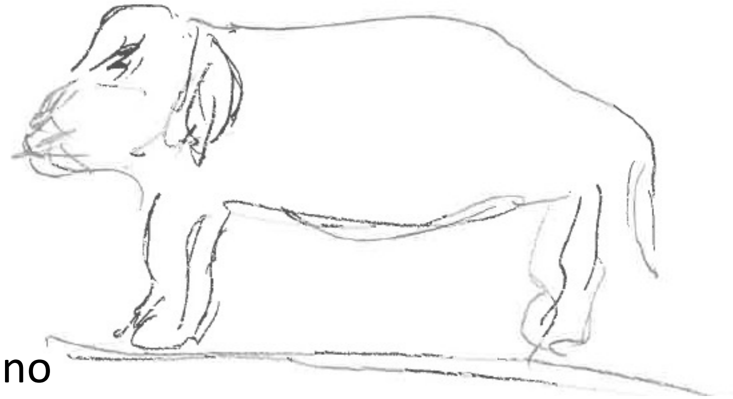
Rhino

Patient 13 (2 months)

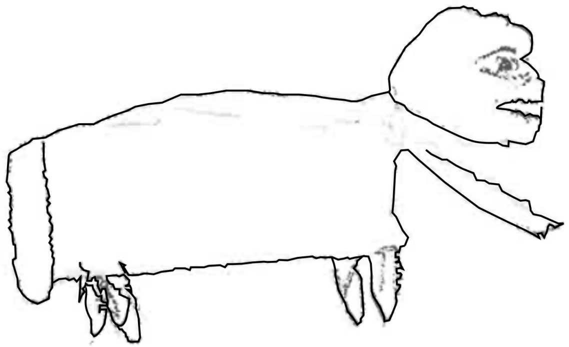

Cat

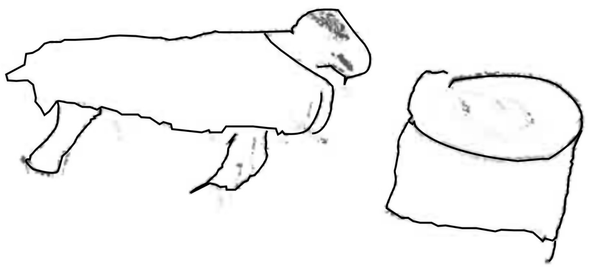

Cow

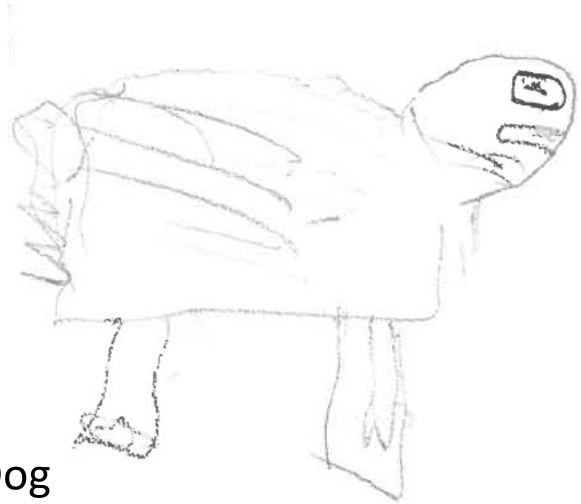

Dog

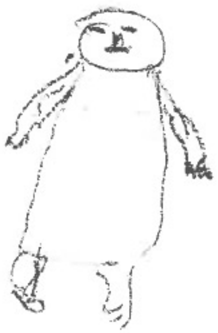

Rabbit

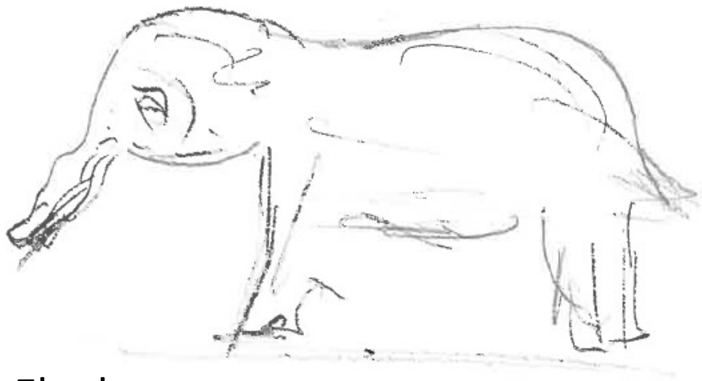

Elephant

Patient 14

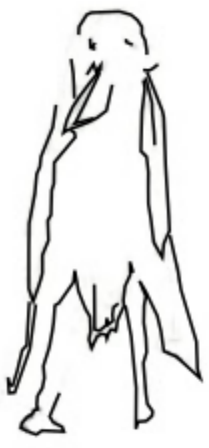

Penguin

Patient 15

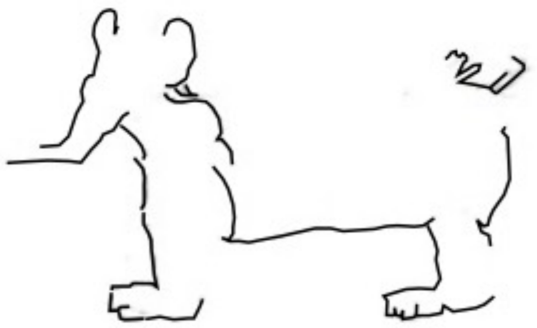

Elephant

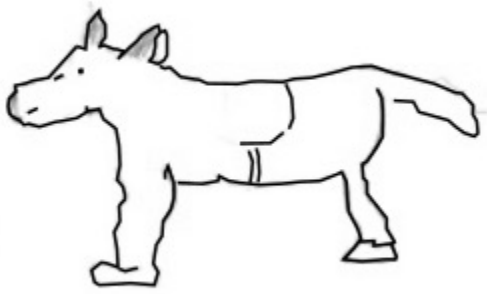

Horse

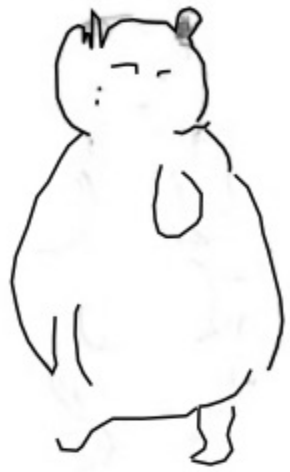

Penguin

Patient 16 (baseline)

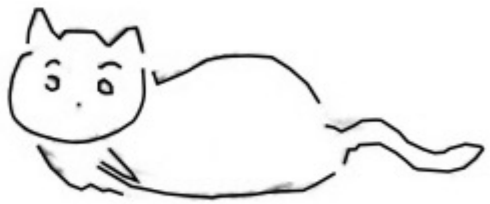

Cat

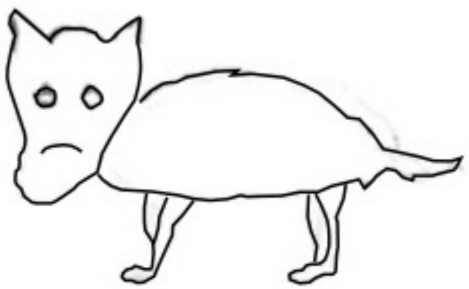

Cow

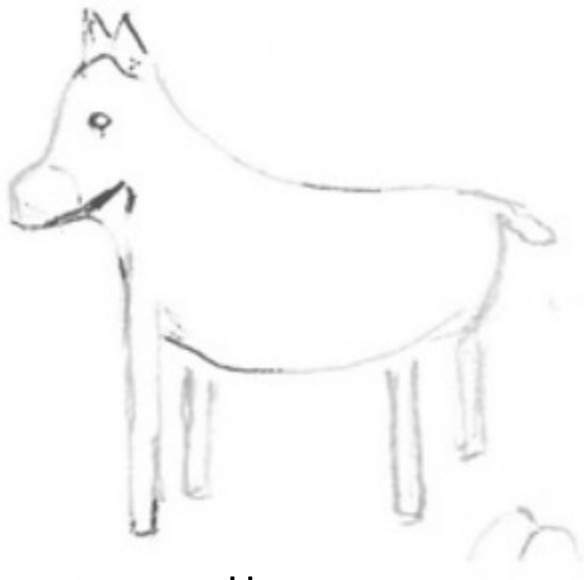

Horse

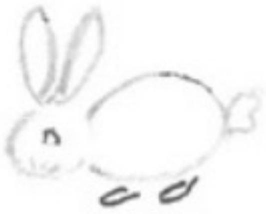

Rabbit

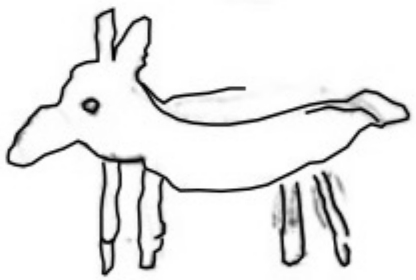

Dog

Patient 16 (3 weeks)

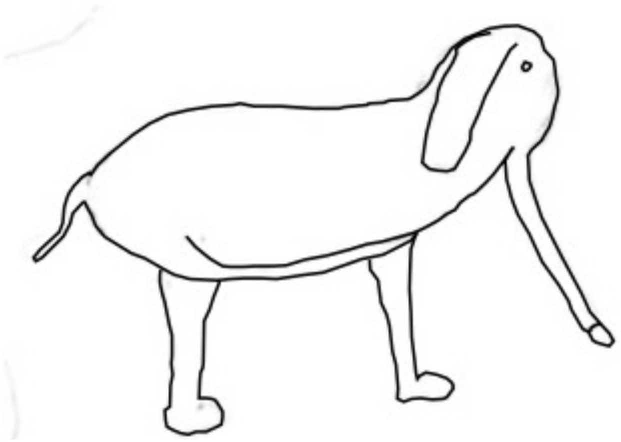

Elephant

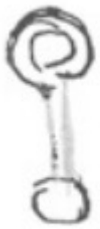

Key

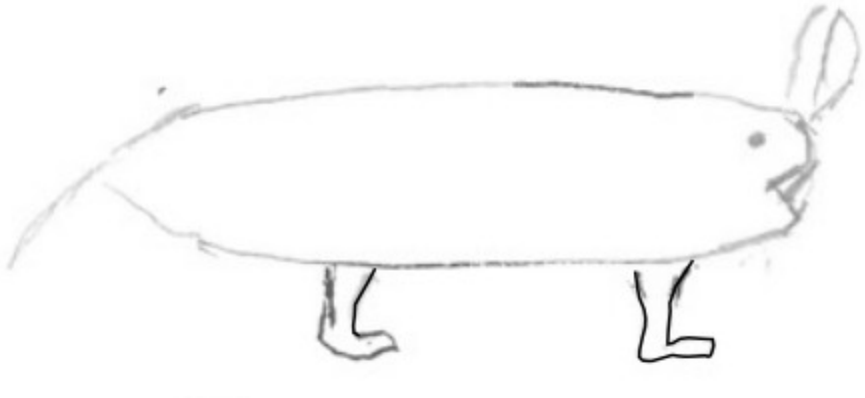

Rhino

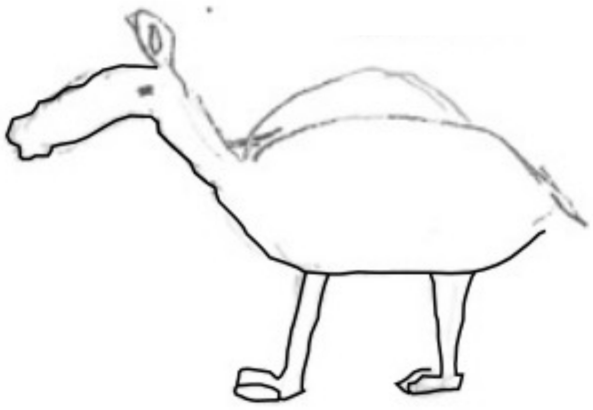

Camel

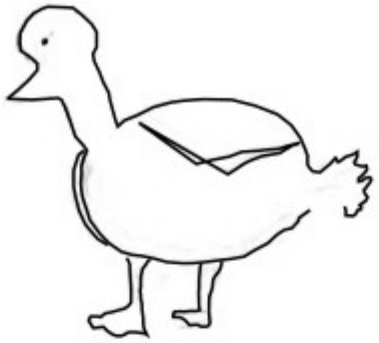

Duck

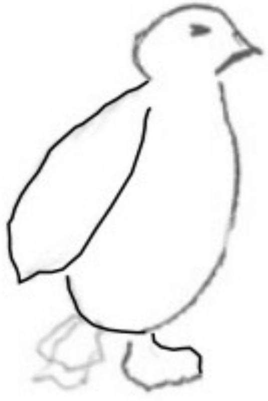

Penguin

Patient 16 (3 weeks)

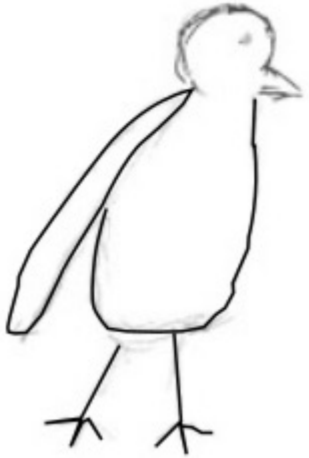

Eagle

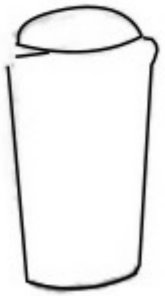

Glass

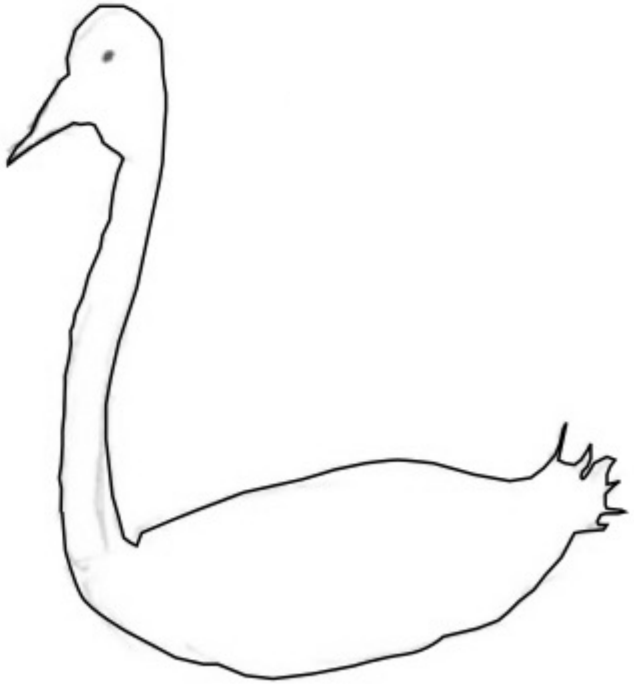

Swan

Patient 17 (baseline)

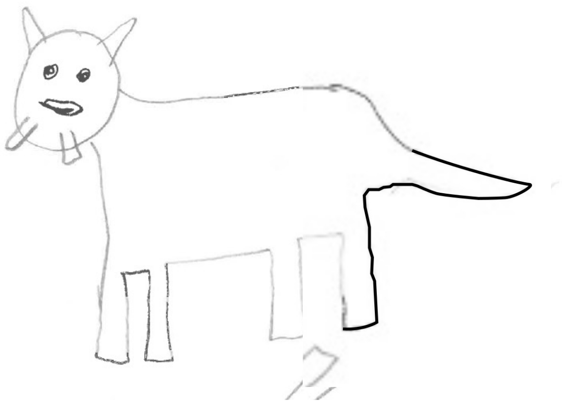

Horse

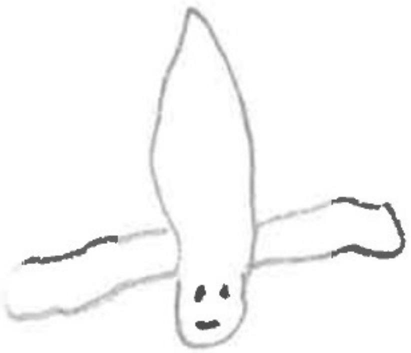

Cat

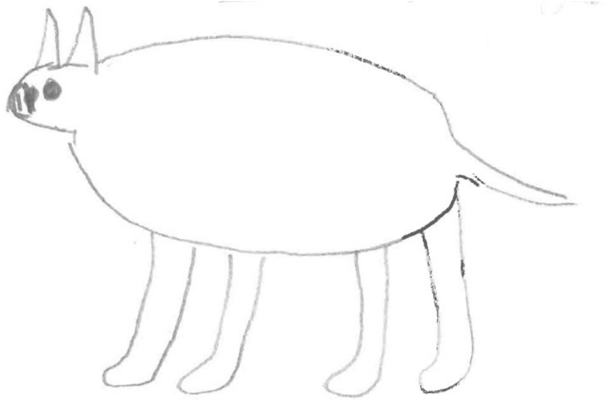

Cow

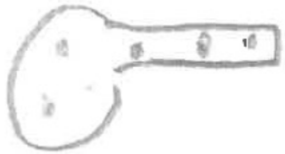

Key

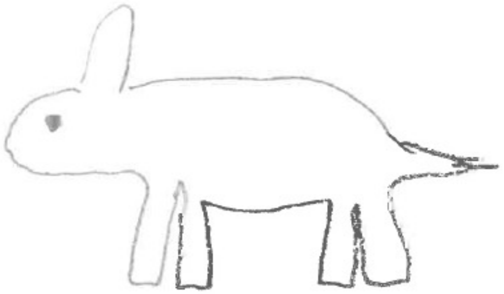

Dog

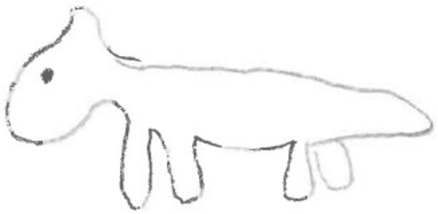

Rabbit

Patient 17 (baseline) continued

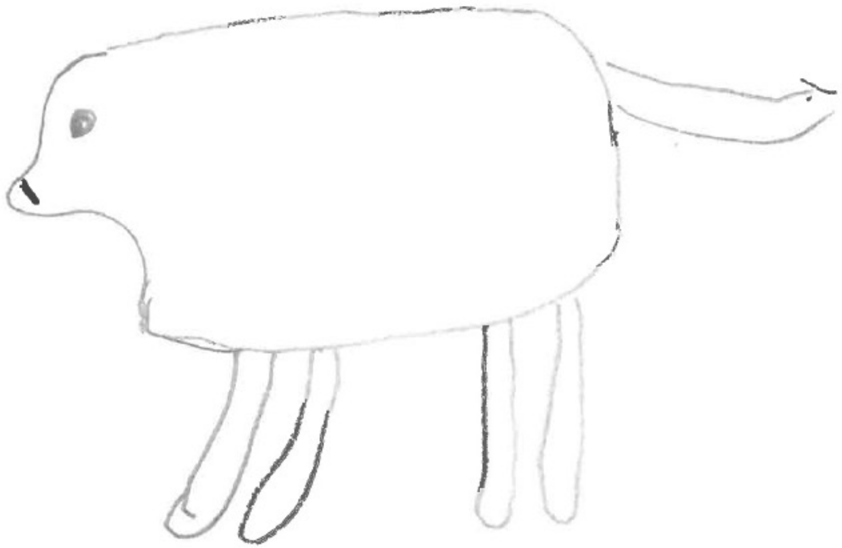

Eagle

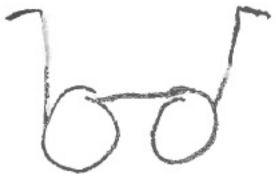

Glass

Patient 18

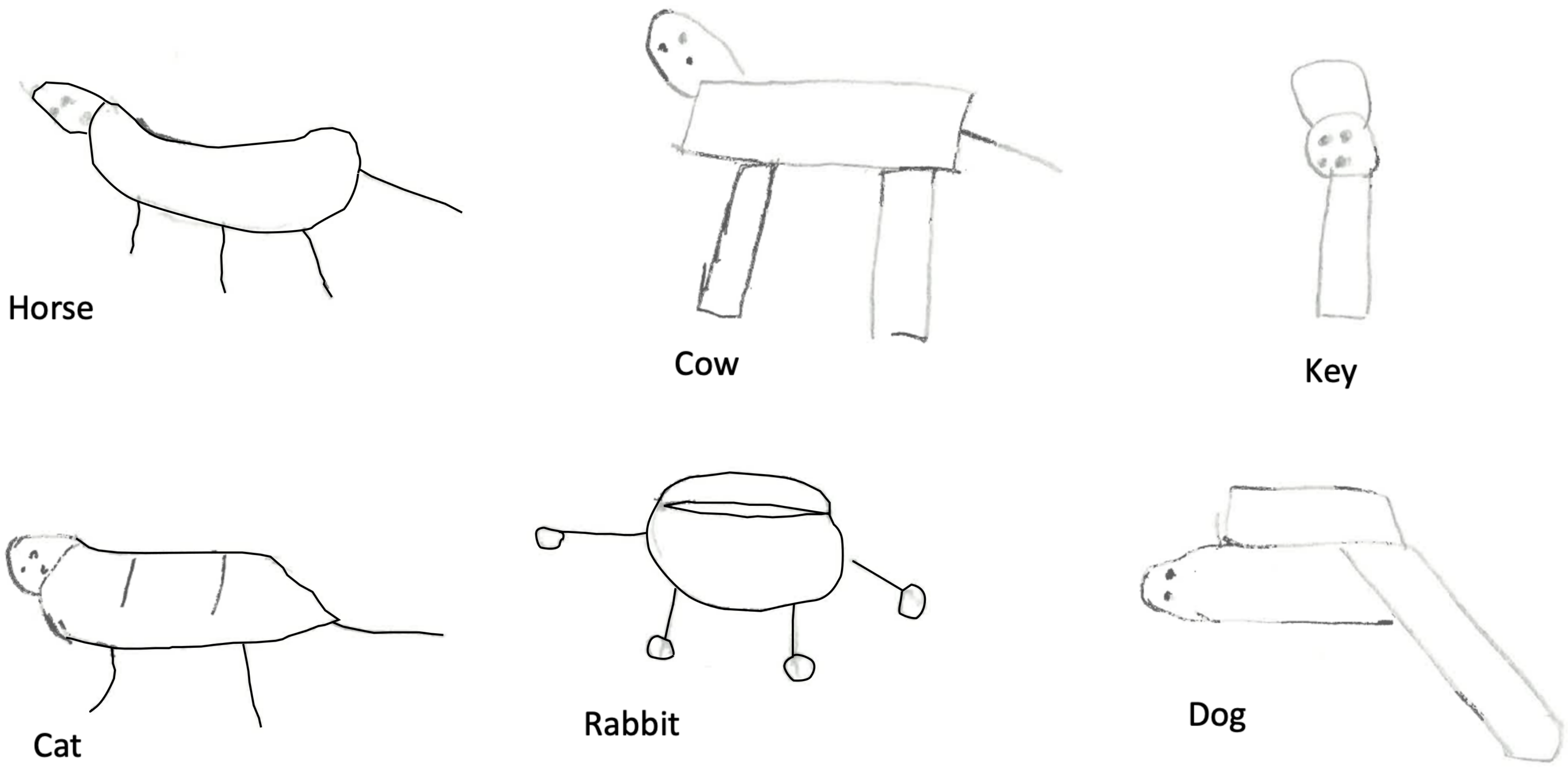

Patient 19

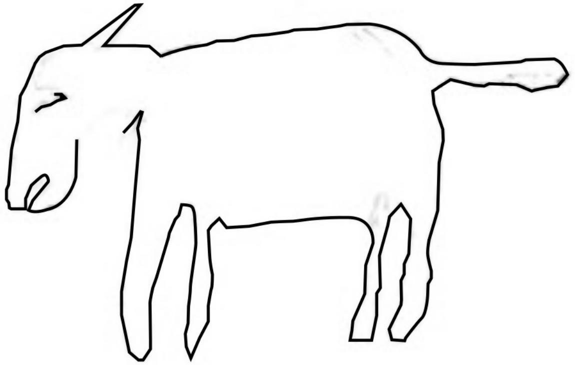

Horse

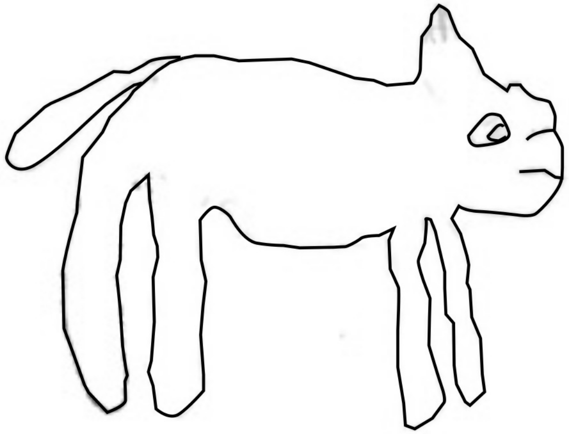

Cat

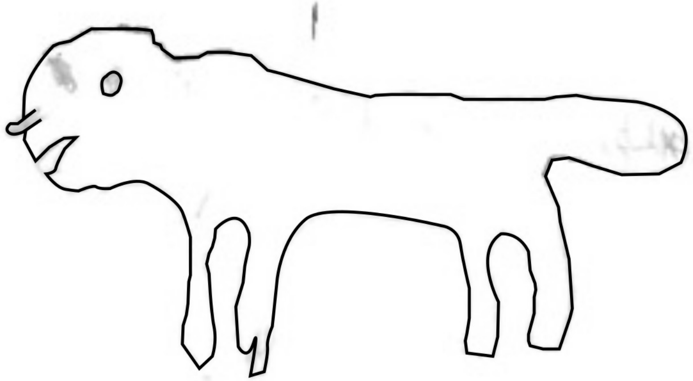

Rabbit

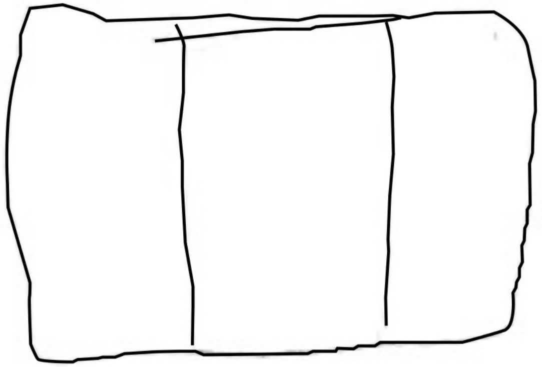

Glass

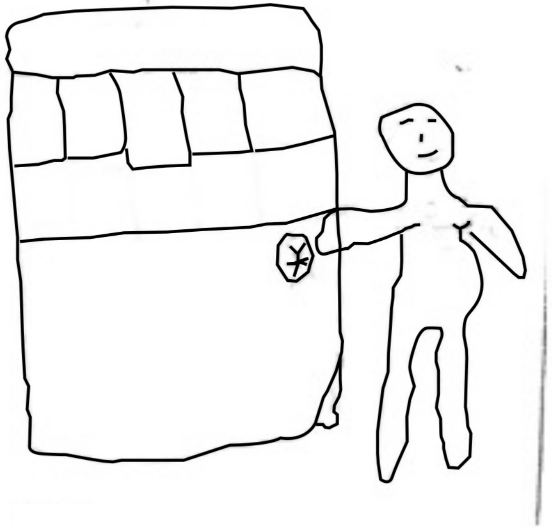

Key

Patient 19 continued

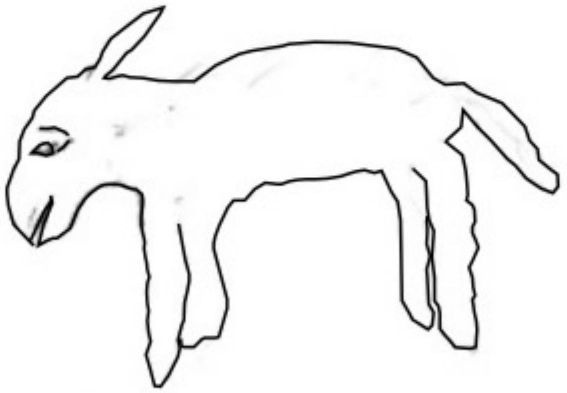

Dog

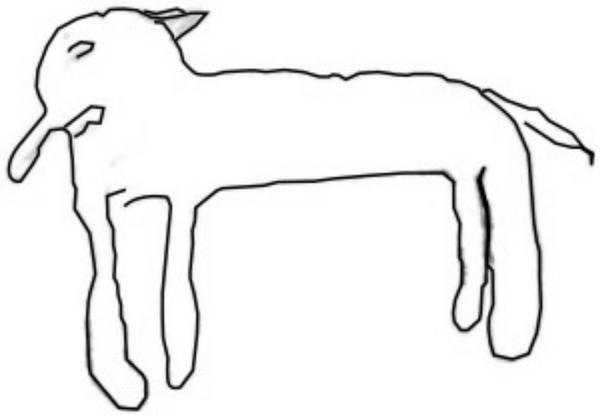

Cow
