# Supplementary material for: Drawing from name in semantic dementia reveals graded object knowledge representations in anterior temporal lobe
Source: Mem Cognit. 2024 May 22;53(1):428–37. doi: 10.3758/s13421-024-01578-9 (PMC11779775; doi:10.3758/s13421-024-01578-9)
Supplement: Supplementary file 2 — Supplementary file2 (DOCX 1019 KB) [file 13421_2024_1578_MOESM2_ESM.docx]

# Supplementary analysis: Exploratory quantitative analysis

# Methods

## Subjects

Analysis compared the 11 contemporary patients (Table 1) with MRI brain scans taken acquired six months of their drawings to the drawings of three age-matched healthy individuals, recruited to produce drawings of living and non-living things in response to the object names, covering the range of objects drawn by the patient cohort.

## Scoring of patient drawings

For exploratory correlation with neuroimaging it is helpful to quantify the amount of information contained within patient drawings with a score. This required the scoring of patients’ residual ability to draw items in comparison to the control participants. This is not a trivial task, as some objects have more characteristic features than others, and patients did not produce exemplars of every item, both because of protocol changes between historical and contemporary cohorts and because more advanced patients were more likely to respond “I don’t know what that is” when asked to draw rarer objects. In order to manage this complexity as best we could, we undertook a multi-stage approach to scoring as described below. These analyses should be viewed as a ‘best exploratory attempt made in good faith’ rather than a definitive method producing reliable and generalisable conclusions.

First, the control subject drawings were assessed by the authors to generate a list of ‘core’ features for each object represented in at least (Supplementary table 1). For the normative drawings, the ‘core’ features were those without which a representation of an animal or object X would not constitute a good example of X, differentiating it from a generic higher-order category template. For example, elephants are quadruped mammals with some features that they share with many other mammals, e.g. four legs, tail, eyes. But they also have the following core features that make them elephants rather than any other mammal: (1) a trunk, (2) large ears, (3) a very large body, and (4) chunky, cylindrical legs. The list for analysis consisted of the features of each object that had been included in the drawings of at least two out of three controls. The tusks on an elephant might be considered ‘core’, but did not meet this criterion (note that large tusks are characteristic of African but not Asian elephants).

Patient drawings were then scored as follows. One point was allocated for each core feature in the list for each object produced by a patient. Thus, patient drawings of elephants were scored out of 4 to yield a proportion of core features represented by patients in their drawings. The proportion of such features for every item attempted for every patient are provided in Supplementary table 2.

Target items differed in difficulty, because of both their differing familiarity and their varying numbers of core features; scores were therefore ratio normalised. The intention of this ratio normalisation was that certain concepts had more weight, such that doing well on a difficult concept affected aggregate scores more than performing above average on several easier items. The reason for this choice was that rarely encountered semantic features are known to be lost first in semantic dementia, while common concepts are preserved until late disease.

We performed normalisation in two ways to assess consistency. In method one, within-sample normalisation, the proportion of core features produced by each patient for each drawing was divided by the average proportion of features produced by our entire cohort for that particular object. For example, 11 patients drew an elephant, with the average proportion of core features represented being 0.232. So, a patient who had drawn 1 out of 4 of these features would be around or very slightly above average, and a normalisation of his or her elephant drawing was scored as 0.25/0.232 = 1.08. However, for a dog (a very commonly encountered animal), the average proportion of core features represented was 0.679. Therefore, a patient producing 1 out of 4 core features would be significantly below average and only score 0.369. Judgements about inclusion of core features were generous rather than strict. For example, any clear indication that an elephant has notably large ears was considered correct; these did not have to be veridical in shape or size.

While suited to the conditions of our assessment, this normalisation method is vulnerable to bias from the fact that patients with more advanced SD were more likely to respond “I don’t know what that is” to the names of rarer items, and thus did not contribute to the normalisation score. We therefore utilised a second method of out-of-sample normalisation. Here, for every item, the proportion of produced features was normalised against a modified population familiarity score produced by Snodgrass & Vanderwart (Snodgrass and Vanderwart, 1980) to correct for the differences in familiarity between objects. Specifically, the Snodgrass familiarity score was rescaled from its original 0-5 range to a range of 0-1. The proportion of features reproduced for each object was then divided by this familiarity score. This meant that, for a given proportion of core features drawn, items less familiar to the general population received higher scores than those that were more familiar to the general population.

Finally, a composite normalisation was performed, applying both methods.

The single subject normalised scores are shown in supplementary table 3. These data were correlated with regionally extracted grey matter volumes, as described in the main text.

# Results

Extracting single-subject grey matter volumes in three regions of interest on each side (Supplementary figure 1, Supplementary figure 2) demonstrated that, across all three normalisation methods, grey matter volume in left inferior temporal gyrus positively correlated with drawing performance (Supplementary figure 3; Pearson r(9)=0.55-0.57, p=0.03-0.04, Spearman rho(9)=0.56-0.69, p=0.01-0.05).

Right inferior temporal gyrus showed a trend towards correlation with drawing performance using the within-sample and combined normalisation methods (r(9)=0.50-0.52, p=0.05-0.06) but not with the Snodgrass (1980) familiarity method alone (r(9)=0.27, p=0.21). Correlations between drawing performance and grey-matter volume of the temporal pole and fusiform gyrus did not reach significance. These non-significant correlations are, however, unsurprising given that there was also substantially less between-subject variability in these regions, with all subjects having severe loss of polar grey matter compared to controls, and most having relatively mild fusiform atrophy.

#
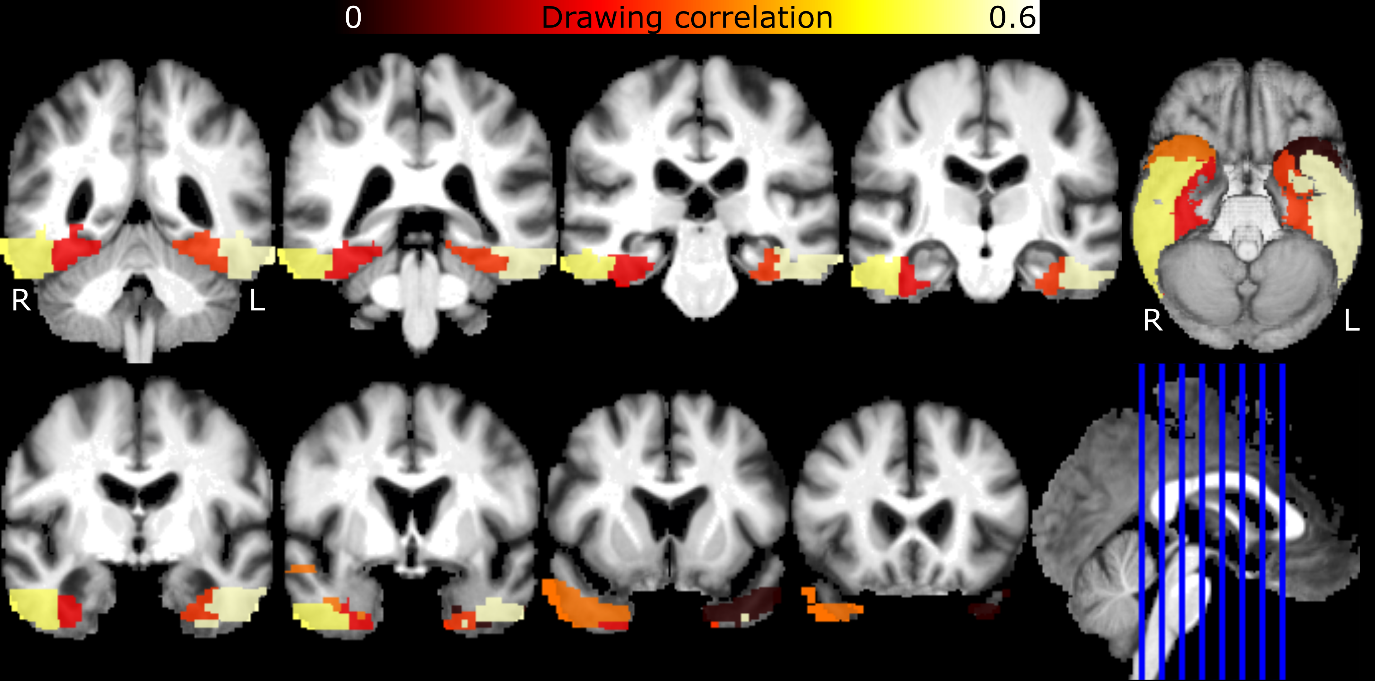
Supplementary Figures

**Supplementary figure 1:** Atlas regions of interest in temporal pole, inferior temporal gyrus and fusiform gyrus, coloured by correlation coefficient against composite drawing score, overlaid on the average brain from the 11 patient and 11 controls used for the study-specific DARTEL template.


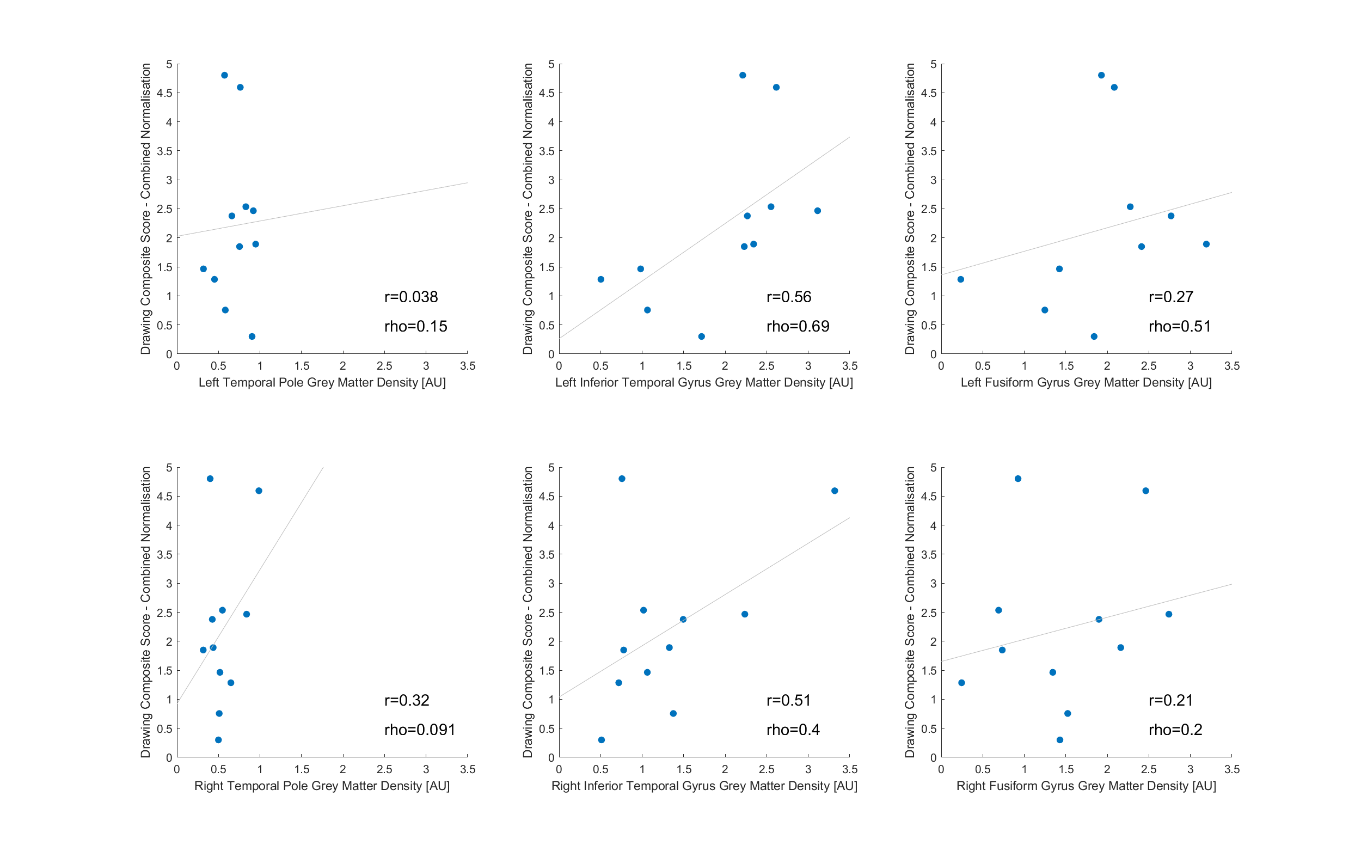


**Supplementary figure 2:** Correlations between composite scores and grey matter density in all ROIs.


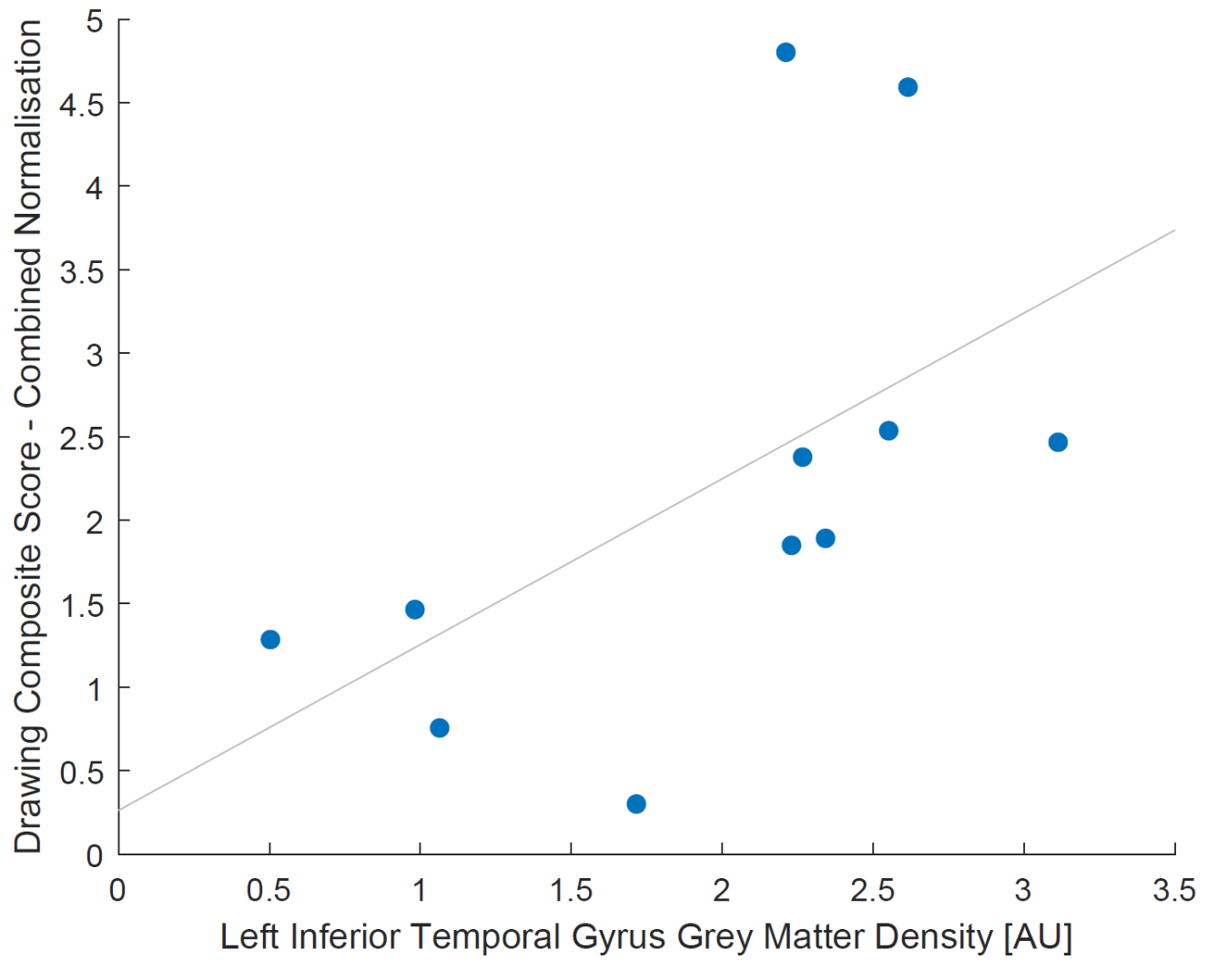


**Supplementary figure 3:** Composite score for the number of specific features contained in patient drawings, against average grey matter density in left inferior temporal gyrus as defined by the AAL atlas. Least squares line shown for Pearson correlation r(9)=0.56, p=0.04. Spearman’s rho(9)=0.69, p=0.01.

# Supplementary tables

| Item | ‘Core’ features | Number of controls producing this feature (/3) |
| --- | --- | --- |
| **Domestic animals:** |  |  |
| Dog | - Nose & snout - Body - Four legs - Tail | - 3 - 3 - 3 - 3 |
| Cat | - Appropriate facial features (nose & whiskers, face resembling pictorial representations of a cat) - Ears - Body - Tail | - 3 - 3 - 3 - 3 |
| Rabbit | - Rounded head - Long ears - Body (round) - Fluffy tail | - 3 - 3 - 3 - 2 |
| **Large animals:** |  |  |
| Horse | - Snout - Ears - Neck - Slender Legs (2 or 4) - Multi-strand tail | - 3 - 2 - 3 - 3 - 3 |
| Cow | - Snout - Fat body - Udder - Tail (cow-like) | - 3 - 3 - 3 - 3 |
| Elephant | - Trunk - Elephant-like ears - Large body - Cylindrical legs | - 3 - 2 - 3 - 3 |
| Camel | - Snout - Neck - Hump (1 or 2) - Single-strand tail | - 3 - 3 - 3 - 3 |
| Rhinoceros | - Horn (1 or 2) - Snout - Heavy Body - Cylindrical Legs (4) - Generic Tail | - 3 - 3 - 3 - 3 - 3 |
| **Birds:** |  |  |
| Duck | - Beak - Neck - Body - Wings - Tail - Legs (2) *or* swimming (then no legs needed) - Duck-like legs (with webbed feet) | - 3 - 2 - 3 - 2 - 3 - 3 - 3 |
| Eagle | - Beak - Wings - Legs *or* drawn flying - Hawk-like tail | - 3 - 3 - 2 - 3 |
| Swan | - Beak - Long Neck - Wings - Tail - Legs with webbed feet *or* swimming | - 3 - 3 - 3 - 3 - 3 |
| Penguin | - Beak - Wings - Upright and cylindrical body - Webbed feet | - 3 - 3 - 3 - 3 |
| **Inanimate objects** |  |  |
| Glass | *Either:*   - Tumbler   *Or:*   - Wine-glass shape top - Shaft - Base | - 2 - 1 - 1 - 1 |
| Key | *Either:*   - Blade & tip - Cuts - Bow   *Or:*   - Bit - Shaft - Bow | - 2 - 2 - 2 - 1 - 1 - 1 |

Supplementary table 1: The core features of each object, determined from analysis of healthy control drawings.

| Patient ID | Date of drawing | Dog | Cat | Rabbit | Horse | Cow | Elephant | Camel | Rhino | Duck | Eagle | Swan | Penguin | Glass | Key |
| --- | --- | --- | --- | --- | --- | --- | --- | --- | --- | --- | --- | --- | --- | --- | --- |
| 1 | Baseline |  | 1.00 |  |  |  |  |  | 1.00 |  |  |  |  |  |  |
| 2 | Baseline |  |  |  |  |  |  | 1.00 | 0.50 |  |  |  |  |  |  |
|  | 2 years |  | 0.75 |  |  |  | 0.25 |  |  | 0.00 |  | 1.00 |  |  |  |
| 3 | Baseline |  |  |  |  |  | 1.00 | 0.00 |  |  |  |  |  |  |  |
| 4 | Baseline |  | 1.00 |  | 1.00 |  |  |  |  | 0.29 |  |  |  |  |  |
|  | 1 year | 0.75 | 0.75 |  | 1.00 |  |  |  |  |  |  |  |  |  |  |
|  | 2 years | 0.75 | 0.50 |  | 1.00 |  |  |  |  |  |  |  |  |  |  |
| 5 | Baseline | 0.75 |  |  |  |  | 0.00 | 0.00 |  | 0.14 |  |  |  |  |  |
|  | Eight months | 0.75 | 0.50 |  |  |  | 0.00 |  |  | 0.14 |  | 0.00 |  |  |  |
| 6 | Baseline | 0.75 |  |  | 0.00 |  |  |  |  | 0.00 |  |  |  |  |  |
| 7 | Baseline |  |  |  |  |  | 0.25 | 1.00 |  |  | 0.33 |  | 0.25 |  |  |
|  | Six months |  |  |  |  |  | 0.00 | 0.75 |  |  | 0.33 |  | 0.50 |  |  |
| 8 | Baseline |  |  | 0.00 | 0.25 |  | 0.25 |  |  | 0.00 |  |  |  |  |  |
| 9 | Baseline | 1.00 |  |  | 0.75 |  | 0.25 |  |  |  |  | 0.60 | 0.25 |  |  |
| 10 | Baseline | 0.75 |  |  |  |  |  | 0.25 |  | 0.29 |  |  | 1.00 |  |  |
| 11 | Baseline | 0.75 |  |  |  | 1.00 | 0.00 |  |  |  |  |  |  |  |  |
| 12 | Baseline | 0.50 |  |  | 0.25 |  | 0.75 | 0.75 |  |  |  |  | 0.75 |  |  |
| 13 | Baseline |  |  |  |  |  |  | 0.50 | 0.00 | 0.00 | 0.00 |  |  | 1.00 |  |
|  | Two months | 0.25 | 0.25 | 0.00 |  | 0.00 | 0.00 |  |  |  |  |  |  |  |  |
|  | Four months |  |  |  | 0.00 |  | 0.00 |  |  |  |  |  |  |  |  |
| 14 | Baseline |  |  |  |  |  |  |  |  |  |  |  | 0.50 |  |  |
| 15 | Baseline |  |  |  | 0.50 |  | 0.00 |  |  |  |  |  | 0.25 |  |  |
| 16 | Baseline | 0.75 | 1.00 | 1.00 | 0.75 | 0.50 |  | 1.00 |  |  |  | 0.60 |  |  |  |
|  | Three weeks |  |  |  |  |  | 0.50 | 1.00 | 0.25 | 1.00 | 1.00 |  | 1.00 | 1.00 | 1.00 |
| 17 | Baseline | 0.75 | 0.00 | 0.00 | 0.25 | 0.50 |  |  |  |  | 0.00 |  |  | 0.00 | 0.33 |
| 18 | Baseline | 0.00 | 0.25 | 0.00 | 0.00 | 0.00 |  |  |  |  |  |  |  |  | 0.00 |
| 19 | Baseline | 1.00 | 0.25 | 0.00 | 0.50 | 0.00 |  |  |  |  |  |  |  | 0.00 | 0.00 |
| Average proportion of core features drawn | 0.68 | 0.57 | 0.17 | 0.48 | 0.33 | 0.23 | 0.63 | 0.44 | 0.21 | 0.33 | 0.55 | 0.56 | 0.50 | 0.33 |  |

Supplementary table 2: The proportion of core features drawn by each individual patient. Blank cells were not attempted.

| Patient ID | Date of drawing assessment | Score, normalised within-sample | Score, normalised to Snodgrass 1980 | Score, dual normalised |
| --- | --- | --- | --- | --- |
| 1 | Baseline | 2.02 | 2.24 | 4.80 |
| 2 | Baseline | 1.37 | 2.02 | 3.80 |
|  | Two years | 0.80 | 0.47 | 1.29 |
| 3 | Baseline | 2.04 | 1.56 | 4.59 |
| 4 | Baseline | 1.74 | 1.04 | 2.51 |
|  | One year | 1.50 | 1.04 | 1.90 |
|  | Two years | 1.36 | 0.94 | 1.72 |
| 5 | Baseline | 0.45 | 0.27 | 0.62 |
|  | Eight months | 0.67 | 0.50 | 0.88 |
| 6 | Baseline | 0.28 | 0.20 | 0.30 |
| 7 | Baseline | 1.03 | 1.09 | 2.38 |
|  | Six months | 0.77 | 1.28 | 1.89 |
| 8 | Baseline | 0.40 | 0.22 | 0.76 |
| 9 | Baseline | 1.14 | 0.85 | 1.85 |
| 10 | Baseline | 1.15 | 1.22 | 2.54 |
| 11 | Baseline | 1.37 | 0.96 | 2.47 |
| 12 | Baseline | 1.40 | 1.30 | 3.04 |
| 13 | Baseline | 0.56 | 0.80 | 0.80 |
|  | Two months | 0.16 | 0.11 | 0.18 |
|  | Four months | 0 | 0 | 0 |
| 14 | Baseline | 0.89 | 1.47 | 2.61 |
| 15 | Baseline | 0.49 | 0.48 | 0.92 |
| 16 | Baseline | 2.25 | 1.36 | 3.77 |
|  | Three weeks | 2.23 | 1.64 | 4.28 |
| 17 | Baseline | 0.52 | 0.32 | 0.76 |
| 18 | Baseline | 0.07 | 0.05 | 0.09 |
| 19 | Baseline | 0.42 | 0.30 | 0.72 |

Supplementary table 3: The normalised proportion scores for all drawings produced by each patient were averaged to create a composite score for each patient. This final composite score was used as a representation of the patient’s semantic knowledge about these objects in further analyses, including correlation with MRI-assessed temporal lobe grey matter volumes.
